# The impact of cerebral oxygen saturation monitoring on perioperative neurocognitive disorders: a meta-analysis and economic analysis

**DOI:** 10.3389/fmed.2026.1677218

**Published:** 2026-01-23

**Authors:** Jiarun Qin, Guoping Wang, Dacheng Gu, Jingjing Li, Jialei Zhang, Mengyuan Ge, Xiaofeng He, Xiaoyan Ma

**Affiliations:** 1 Department of Anesthesiology, Changzhi People's Hospital, Changzhi, China; 2 Department of Otolaryngology, Changzhi People's Hospital, Changzhi, China; 3 Institute of Evidence-Based Medicine, Heping Hospital Affiliated to Changzhi Medical College, Changzhi, China; 4 Department of Pain Treatment, Changzhi People's Hospital, Changzhi, China

**Keywords:** cerebral oxygen saturation, cognitive dysfunction, delirium, economic evaluation, meta-analysis, neuropsychological tests, perioperative neurocognitive disorders, postoperative cognitive complications

## Abstract

**Background:**

Inadequate intraoperative cerebral oxygen supply is one of the inciting causes of postoperative cognitive disturbances. Numerous studies have investigated the association between regional cerebral oxygen saturation (rScO_2_) monitoring and postoperative cognitive dysfunction. However, results are inconsistent, owing to differences in surgery type, patient population, and monitoring protocols. Therefore, we conducted a meta-analysis to comprehensively evaluate the association between rScO_2_ monitoring and the incidence of postoperative neurocognitive disorders.

**Methods:**

A comprehensive literature search was conducted across multiple databases from their inception to June 2025 to identify randomized controlled trials (RCTs) that compared the impact of rScO_2_ monitoring versus no monitoring on cognitive function. The primary outcome was the incidence of perioperative neurocognitive disorders (PNDs). Secondary outcomes were the incidences of postoperative cognitive dysfunction (POCD) and postoperative delirium (POD), as well as the economic indicators of the number needed to treat (NNT) and cost–benefit ratio (CBR).

**Results:**

A total of 28 RCTs were included. Overall, we found that intraoperative rScO_2_ monitoring significantly reduced the incidence risk of PND (relative risk [RR] = 0.47, 95% confidence interval [CI]: 0.41, 0.54), POCD (RR = 0.47, 95% CI: 0.39, 0.57), and POD (RR = 0.45, 95% CI: 0.35, 0.57). Subgroup analyses based on surgery type (cardiac, orthopedic, abdominal, and others) demonstrated consistent protective effects of monitoring. Sensitivity analyses using leave-one-out analysis, excluding Chinese-language publications, low-quality studies, and studies with a baseline rScO_2_ < 80%, confirmed the robustness of results. The economic evaluation showed that rScO_2_ monitoring is both clinically beneficial and cost-effective, as reflected in the low NNT values and favorable CBRs, which indicated that the cost of prevention is substantially lower than that of managing complications.

**Conclusion:**

Intraoperative rScO_2_ monitoring significantly reduces the incidence of PND, including POCD and POD. Consistent protective effects were observed across a wide range of surgery types, demonstrating its broad clinical applicability. Furthermore, its favorable cost–benefit profile demonstrated that the prevention of neurocognitive complications has a substantially lower cost than the estimated economic burden of managing these complications. Widespread adoption of rScO_2_ monitoring is recommended to improve postoperative cognitive outcomes.

## Introduction

In modern anesthesiology, perioperative neurocognitive disorders (PND) have become an important clinical concern. The term PND was introduced in 2018 to replace the previous term, postoperative cognitive dysfunction (POCD), and now encompasses both POCD and postoperative delirium (POD) (1). We have divided PND into POCD and POD in this study according to the varied follow-up durations and content. POCD is a common central nervous system complication that is characterized by various symptoms, such as confusion, anxiety, personality changes, and memory impairment, which profoundly affect postoperative recovery and quality of life (2). By contrast, POD is an acute and fluctuating mental state characterized by inattention, disorganized thinking, and altered consciousness, and occurs without prior neurocognitive disorders or coma (3). PND not only prolongs hospital stays and increases the risk of other postoperative complications but also raises mortality rates and negatively impacts long-term quality of life. Thus, identifying approaches to reduce the incidence of PND is crucial.

The exact cause of PND remains unclear, although studies have suggested a potential association with general anesthesia (4). Recently, Glumac et al. (5) conducted a 4-year follow-up study and reported that a single preoperative administration of dexamethasone significantly alleviates the inflammatory response caused by cardiac surgery and reduces the incidence and severity of postoperative cognitive impairment. This indicates a possible link between inflammation and POCD, with inflammatory responses likely playing a key role in the development of PND. Similar mechanisms have been explored in previous meta-analyses (6). Cerebral oxygen saturation is a critical indicator of the brain’s oxygen supply–demand balance (7). Intraoperative factors, such as excessive intraoperative blood loss, hypoxemia, and hypotension, can lead to inadequate cerebral perfusion, causing decreased regional cerebral oxygen saturation (rScO_2_), which can disrupt cerebral oxygen metabolism and trigger neurological complications. A recent study by Colak et al. found that prolonged desaturation of rScO_2_ is an important predictor of cognitive decline (8). During surgery, the effects of anesthesia on cerebral oxygen balance may contribute significantly to an increased risk of developing postoperative neurological complications (9). Real-time monitoring of rScO_2_ provides immediate information on the brain’s oxygen supply and is crucial for preventing PND. Compared with other monitoring techniques, rScO_2_ measurement is simpler and more practical in clinical settings. By monitoring rScO_2_, anesthesiologists can better manage anesthetic depth and hemodynamics, which enables timely interventions that can reduce the incidence of PND.

Although numerous studies have investigated the role of rScO_2_ monitoring in different surgeries, findings are inconsistent. Therefore, this meta-analysis aimed to evaluate the impact of intraoperative rScO_2_ monitoring on the incidence of PND, POCD, and POD. Additionally, we aimed to determine the effectiveness of rScO_2_ monitoring in improving postoperative cognitive outcomes and assess its economic benefits via cost-effectiveness analysis.

## Methods

This study was performed according to the statement on preferred reporting items for systematic reviews and meta-analyses (S1 PRISMA Checklist in Supplementary material) (10).

### Inclusion and exclusion criteria

Inclusion criteria for studies were as follows: (1) adult patients undergoing surgery under general or spinal anesthesia, American Society of Anesthesiologists classification I–III; (2) use of rScO_2_ monitoring during surgery; (3) neuropsychological testing performed preoperatively and 1 week postoperatively to assess the occurrence of PND; and (4) studies with a clearly defined sample size. Exclusion criteria were as follows: (1) studies lacking original data or with incomplete information, preventing data extraction; (2) patients with pre-existing conditions that could affect postoperative cognitive function, such as dementia, stroke, other central nervous system diseases, a history of alcohol or drug abuse, multiple injuries, or traumatic brain injuries; (3) patients unable to undergo normal verbal communication because of language barriers or other reasons, which could interfere with pre- and postoperative neuropsychological testing; and (4) secondary studies such as meta-analyses, case reports, reviews, abstracts, letters, and non-original research studies. Strict inclusion and exclusion criteria were applied to ensure homogeneity among study subjects and reproducibility of results. This allowed for a reliable evaluation of the impact of rScO_2_ monitoring on postoperative cognitive function.

### Search strategy

The study subjects were patients undergoing general or spinal anesthesia, regardless of surgery type. A comprehensive search was conducted using the following databases: CNKI, Wanfang, VIP, PubMed, Web of Science, Cochrane, and Embase. The search aimed to identify all randomized controlled trials (RCTs) that met the inclusion criteria, covering studies from database inception to June 2025 to ensure the inclusion of the latest findings. Only human studies were included; animal studies were excluded. The search terms were as follows: cerebral oxygen saturation, rScO_2_, near-infrared spectroscopy (NIRS), perioperative neurocognitive disorders, postoperative cognitive dysfunction, and postoperative delirium.

### Data extraction

During the data extraction phase, a data extraction form was designed. Two researchers (JQ and DG) independently screened all articles according to the inclusion and exclusion criteria, and subsequently extracted relevant data for statistical analysis. If the two researchers had a disagreement that could not be resolved, a third researcher (JL) re-extracted the data for further analysis. All extracted data were cross-checked and confirmed by all three researchers (JQ, DG, and JL) to ensure accuracy. If there were any ambiguities or disputes regarding the data in any article, the original authors were contacted to obtain accurate original data. Extracted information included: first author, publication year, sample size, event count in the experimental and control groups, surgery type, assessment methods, and primary outcome measures.

### Outcome measures and quality assessment

The primary outcome was the incidence of PND, which was used to evaluate the incidence of PND across different surgical groups. To assess the risk of bias in the included studies, we used the risk bias assessment tool Risk of Bias 2 (11) (RoB 2), as recommended by Cochrane for conducting a systematic review of RCTs. Each assessment result was classified as “low risk,” “some concern,” or “high risk.” The assessment work was completed independently by two researchers (JQ and DG). If there were any inconsistencies, the final assessment result was determined following discussion with a third researcher (JL).

### Statistical analysis

Statistical analyses were conducted using relative risk (RR) with corresponding 95% confidence intervals (CI). Heterogeneity among studies was assessed using the Q test and the *I^2^* statistic (12). A random-effects model was used for all analyses for two main reasons: (1) the Q test is characterized by low statistical power for between-study heterogeneity, which is especially relevant when few studies are available; and (2) the random-effects model is a more conservative choice when heterogeneity is present, whereas it reduces to the fixed effects model when heterogeneity is absent. *p* < 0.05 was considered statistically significant. Subgroup analyses were conducted to assess the effects of the intervention in different surgical categories, including cardiac surgery, orthopedic surgery, abdominal surgery, and other types of surgery. Supplementary subgroup analyses considered cognitive assessment tools, follow-up periods, and intervention thresholds for rScO_2_. Sensitivity analysis was performed using the following four steps: (1) one-by-one elimination method, whereby one study was eliminated at a time to observe changes in effect size; (2) exclusion of Chinese-language publications to assess potential language bias; (3) exclusion of low-quality studies (classified as “high risk” or “some concerns” using the RoB 2 tool) to explore their impact on the overall results; and (4) exclusion of studies in which the intervention was applied only when intraoperative rScO_2_ dropped to less than 80% of baseline to evaluate the impact of delayed or reactive interventions and ensure consistency in the monitoring thresholds across studies. Publication bias was analyzed using Begg’s funnel plot (13) and Egger’s test (14) when more than 10 studies were available. If publication bias was detected, the “trim-and-fill method” was used to adjust the results (15). The quality of evidence for each outcome was evaluated using the Grading of Recommendations, Assessment, Development, and Evaluations (GRADE) framework (16), which allows transparent and systematic ratings of evidence quality based on various factors, such as risk of bias, inconsistency, indirectness, imprecision, and other considerations. To further evaluate the clinical and economic benefits of rScO_2_ monitoring in preventing PND, two additional metrics were calculated: (1) number needed to treat (NNT) for therapeutic effect (17), which was defined as the reciprocal of the absolute difference in the incidence of PND between the intervention and control groups, calculated using the following formula: NNT = 1/attributable risk reduction (ARR); ARR represents the difference in the incidence of PND between the two groups; and (2) cost–benefit ratio (CBR) (18), which was calculated as the ratio of the total intervention cost required to prevent one case of PND to the economic loss that could have been avoided for that case using the formula, CBR = (NNT × monitoring cost per patient) / total cost reduction for each case of neurocognitive-related complications. All statistical analyses were performed using Stata version 18.0 (Stata Corporation, College Station, TX, United States).

## Results

### Literature search results

**Figure 1:**
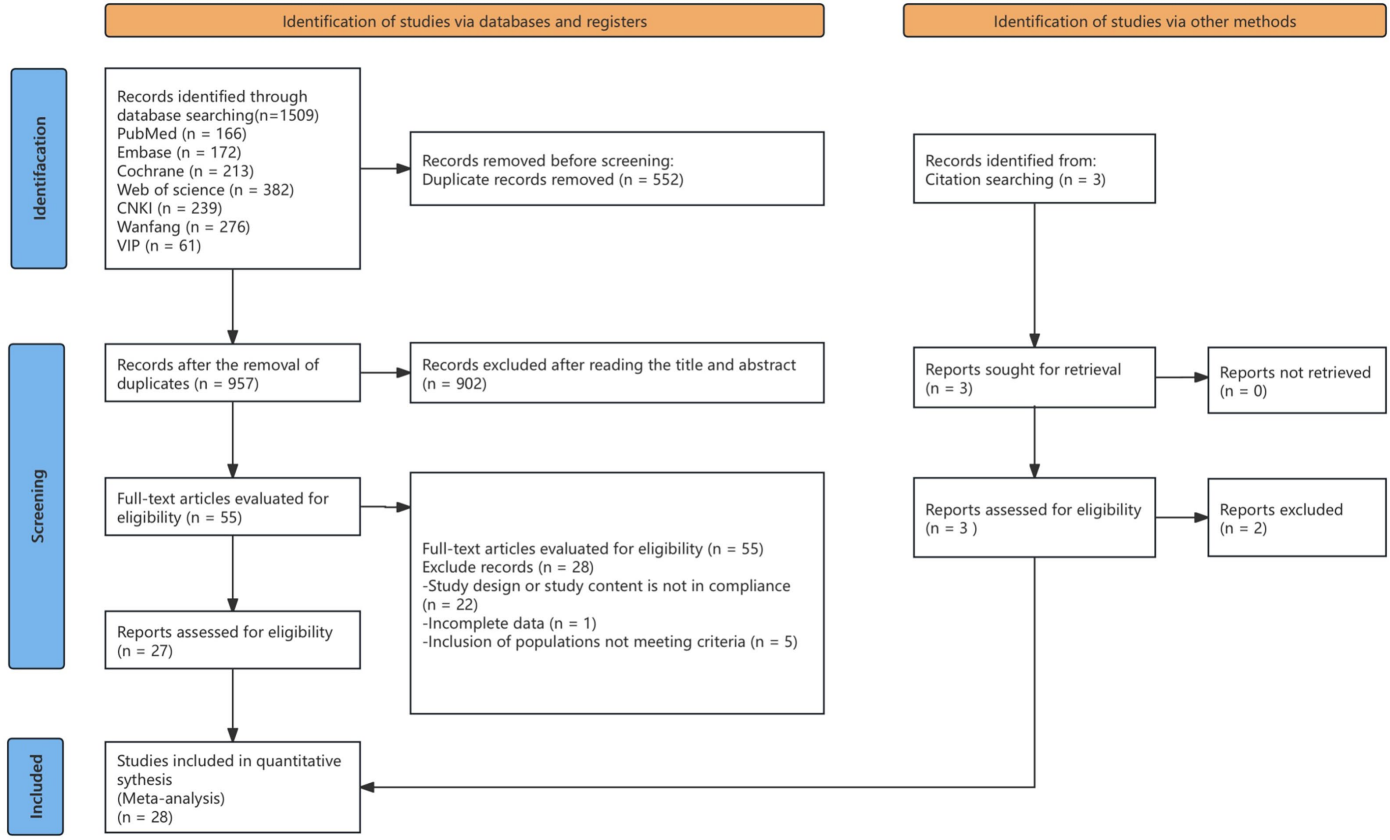
Flow diagram for searching included articles.

**Figure 2:**
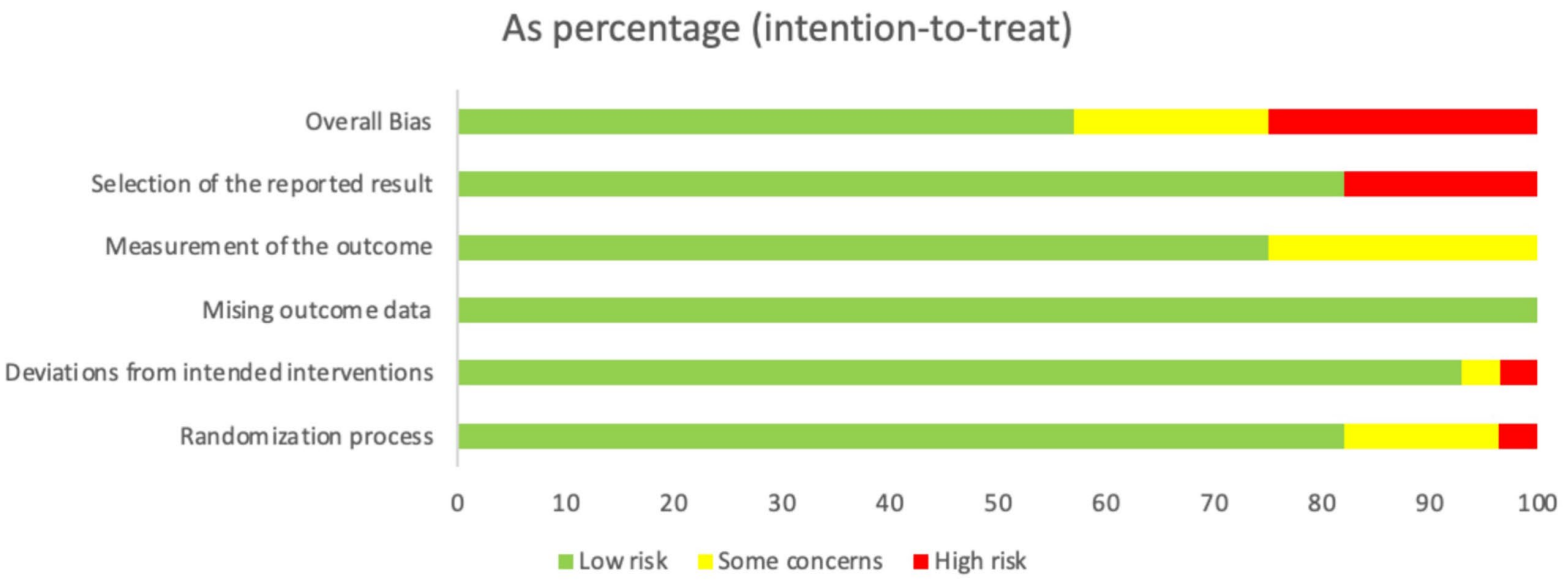
The proportion of each methodological quality item.

**Figure 3:**
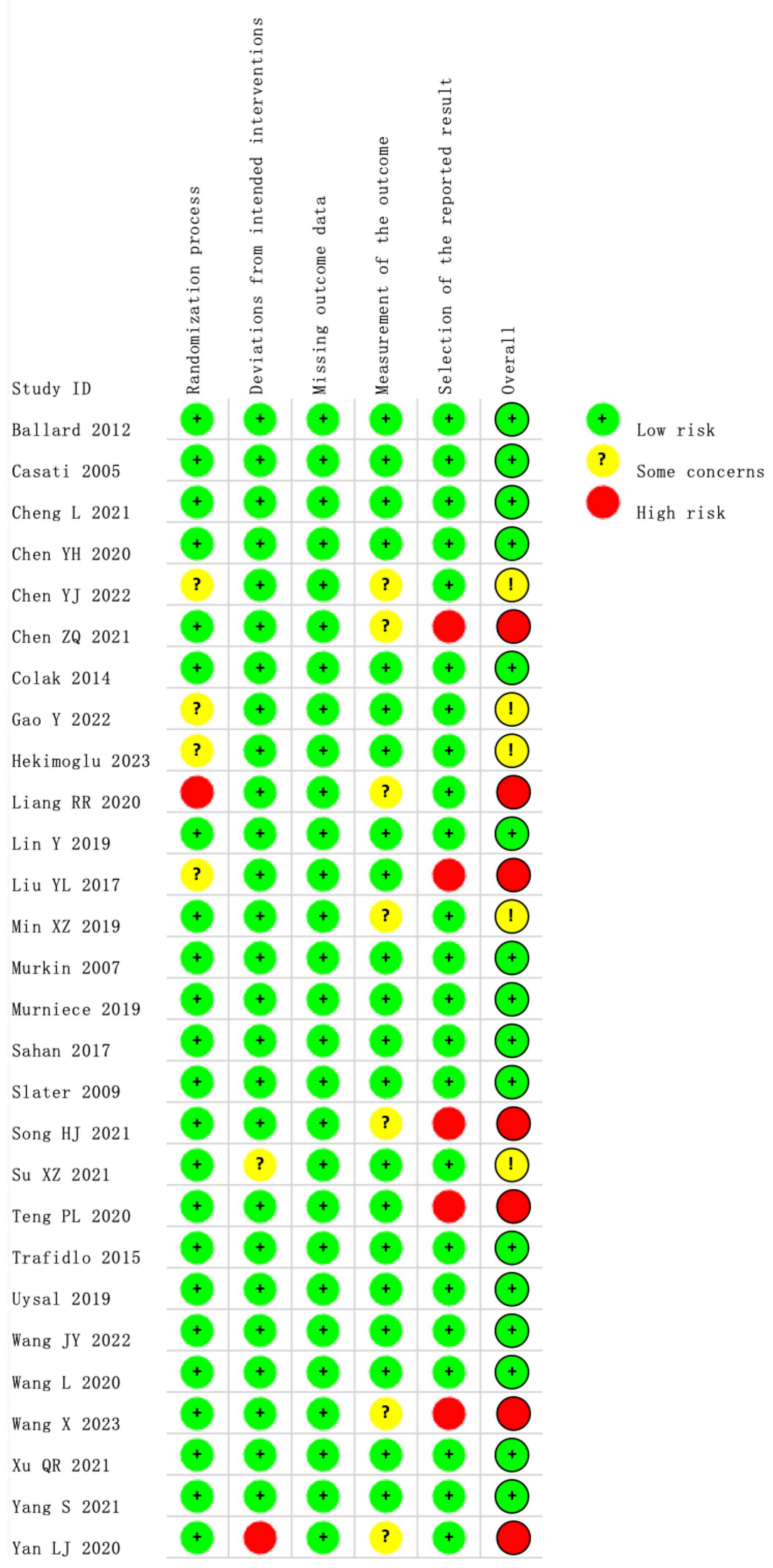
The methodological quality assessment.

Our search of the databases and registers identified 1,509 articles. After removing 552 duplicates, 957 records remained. Following title and abstract review, 902 records were excluded as irrelevant, and 55 records advanced to full-text evaluation. Of these, 28 articles were excluded because of inappropriate study design or content (*n* = 22), incomplete data (*n* = 1), or ineligible populations (*n* = 5). An additional three reports from other sources were assessed, of which two were excluded. Ultimately, 28 articles were included in the meta-analysis (8, 19–45) (Figure 1), comprising 2,824 patients, of whom 1,401 patients were in the experimental group and 1,423 were in the control group. The evaluation of study quality using the RoB2 tool is provided in Supplementary Table 1. The proportion of each methodological quality item is presented in Figure 2, and the methodological quality assessment of the included studies is shown in Figure 3.

### Characteristics of included studies

There were no significant differences in the baseline characteristics of the patients across the included studies, which included age, sex, body mass index, and other baseline variables (Table 1). Postoperative follow-ups were conducted to assess cognitive function indicators. PNDs encompass all neurocognitive disorders related to surgery and anesthesia and include various cognitive problems that may arise during the perioperative period. PND can manifest as short-term or long-term cognitive impairment at different postoperative stages and aims to reflect the broad spectrum of cognitive dysfunctions during the perioperative period, with a particular focus on two common postoperative cognitive issues: POD and POCD. POCD is a persistent cognitive impairment that affects long-term recovery, whereas POD is an acute, early postoperative cognitive disorder with short-lived and fluctuating symptoms.

**Table 1:** Basic characteristics of included studies.

The first author (Year)	Experimental group		Control group		Surgical type	Outcome (time point)	Evaluation	Main baseline characteristics (age, sex, BMI)	rScO₂ intervention threshold (%)
	Sample size	Incidence (%)	Sample Size	Incidence (%)					
Wang L (19) (2020)	60	11 (18.3%)	60	22 (36.7%)	Orthopedics surgery	POD (7 day)	CAM	No significant difference	>90%
Cheng L (20) (2021)	37	2 (5.4%)	39	10 (25.6%)	Abdominal surgery	POD (3 day)	CAM	No significant difference	>65%
Chen YH (21) (2020)	30	1 (3.3%)	30	8 (26.7%)	Orthopedics surgery	POD (7 day)	CAM	No significant difference	>80%
Yan LJ (22) (2020)	53	10 (18.9%)	54	24 (44.4%)	Other surgeries	POD (3 day)	CAM	No significant difference	>80%
Teng PL (23) (2020)	30	7 (23.3%)	30	16 (53.3%)	Other surgeries	POD (5 day)	CAM	No significant difference	>90%
Xu QR (24) (2021)	59	9 (15.3%)	58	17 (29.3%)	Orthopedics surgery	POCD (7 day)	MoCA	No significant difference	>90%
Chen ZQ (25) (2021)	32	4 (12.5%)	32	28 (87.5%)	Other surgeries	POCD (4 day)	MMSE	No significant difference	>89%
Su XZ (26) (2021)	28	5 (17.9%)	29	13 (44.8%)	Other surgeries	POCD (5 day)	MoCA	No significant difference	>87%
Wang JY (27) (2022)	78	8 (10.3%)	81	28 (34.6%)	Other surgeries	POD (7 day)	CAM	No significant difference	>80%
Ballard (28) (2012)	34	6 (17.6%)	38	23 (60.5%)	Orthopedics surgery	POCD (3 month)	MMSE	No significant difference	NR
Colak (8) (2014)	88	25 (28.4%)	93	48 (51.6%)	Cardiac surgery	POCD (7 day)	MMSE	No significant difference	>80%
Murkin (29) (2007)	100	11 (11%)	100	18 (18%)	Cardiac surgery	POCD (1 month)	MMSE	No significant difference	>75%
Slater (30) (2009)	19	7 (37%)	21	10 (45%)	Cardiac surgery	POD (7 day)	DRS	No significant difference	>80%
Trafidlo (31) (2015)	13	2 (15.4%)	30	12 (40%)	Orthopedics surgery	POCD (1 month)	MMSE	No significant difference	>80%
Murniece (32) (2019)	23	3 (13%)	11	4 (36.4%)	Orthopedics surgery	POCD (2 day)	MoCA	No significant difference	>80%
Uysal (33) (2019)	59	2 (3.4%)	66	7 (10.6%)	Cardiac surgery	POCD (3 month)	MMSE	No significant difference	>60%
Chen YJ (34) (2022)	30	6 (20%)	30	22 (73.3%)	Orthopedics surgery	POCD (7 day)	MMSE	No significant difference	>80%
Gao Y (35) (2022)	38	3 (7.9%)	37	11 (29.7%)	Cardiac surgery	POD (2 day)	DRS	No significant difference	>80%
Min XZ (36) (2019)	30	5 (16.7%)	30	13 (43.3%)	Cardiac surgery	POCD (7 day)	MMSE	No significant difference	>89%
Wang X (37) (2023)	53	4 (7.5%)	53	8 (15.1%)	Orthopedics surgery	POCD (7 day)	MMSE	No significant difference	>80%
Sahan (38) (2017)	19	7 (36.8%)	21	9 (42.9%)	Cardiac surgery	POCD (7 day)	MMSE	No significant difference	>80%
Yang S (39) (2021)	12	2 (16.7%)	14	4 (28.6%)	Orthopedics surgery	POCD (7 day)	MoCA	No significant difference	>80%
Liang RR (40) (2020)	34	7 (20.6%)	21	10 (47.6%)	Abdominal surgery	POCD (7 day)	MoCA	No significant difference	>85%
Liu YL (41) (2017)	137	20 (14.6%)	131	37 (28.2%)	Abdominal surgery	POD (7 day)	CAM-ICU	No significant difference	>80%
Lin Y (42) (2019)	63	1 (1.6%)	74	2 (2.7%)	Cardiac surgery	POD (7 day)	CAM-ICU	No significant difference	>70%
Song HJ (43) (2021)	30	2 (6.7%)	30	8 (26.7%)	Orthopedics surgery	POCD (7 day)	MoCA	No significant difference	>55%
Casati (44) (2005)	56	20 (35.7%)	66	36 (54.5%)	Abdominal surgery	POCD (7 day)	MMSE	No significant difference	>75%
Hekimoglu (45) (2023)	50	19 (38%)	50	41 (82%)	Other surgeries	POCD (5 day)	MMSE	No significant difference	>80%

When cognitive function assessment tools (such as MMSE and MoCA) are used on the 4th day after the operation and later, the results are classified as POCD. The results are classified as POD only when a definite delirium screening tool such as CAM or CAM-ICU is used within 7 days after the operation. Although some studies used MMSE/MoCA to assess cognitive status in the early postoperative period, due to the characteristics of the tools not being suitable for diagnosing POD, they were uniformly classified under the category of POCD.

Intervention thresholds refer to the criteria used in each study to initiate intraoperative measures in response to rScO₂ desaturation.

POCD: Postoperative cognitive dysfunction refers to a decline in learning ability, memory, executive function, attention, and other cognitive functions in patients after surgery and anesthesia compared to their preoperative state.

POD: Postoperative delirium is an acute cognitive impairment characterized primarily by memory disturbances and impaired consciousness. It typically occurs in the early postoperative period, within 1 to 3 days after surgery, and generally lasts only a few days.

NR: not reported.

The commonly used methods to assess PND (Postoperative Neurocognitive Disorders) include the Mini-Mental State Examination [MMSE (54)], the Montreal Cognitive Assessment [MoCA (55)], and the Confusion Assessment Method for the ICU [CAM-ICU (56)] and the Delirium Rating Scale [DRS (57)].

The commonly used methods to assess PND (Postoperative Neurocognitive Disorders) include the Mini-Mental State Examination (MMSE), the Montreal Cognitive Assessment (MoCA), and the Confusion Assessment Method for the ICU (CAM-ICU).

The MoCA scale is designed to address the characteristics of mild cognitive impairment, with a focus on memory impairment as the main manifestation. It includes an extended delayed recall time and adjusts the scoring weight accordingly, making it more targeted to the specific features of mild cognitive impairment.

The Confusion Assessment Method (CAM) is used as a clinical aid in diagnosing delirium in the elderly. It has a high sensitivity (94–100%) and specificity (90–95%). The assessment is quick, easy to administer, and simple to operate, with good reliability and validity.

The Delirium Rating Scale (DRS), especially its revised version DRS-R-98, is a validated instrument used to evaluate the severity and symptom profile of delirium. It assesses multiple cognitive and behavioral domains, and is frequently employed in both clinical and research settings to quantify delirium severity and monitor changes over time.

PND, POD, and POCD reflect varying degrees and durations of the impact of surgery and anesthesia on cognitive function, highlighting the need for appropriate monitoring and management at different postoperative stages, and therefore we performed analyses for PND, POCD, and POD separately. This allowed us to examine the characteristics of cognitive dysfunction at different postoperative stages.

### Meta-analysis results

#### Perioperative neurocognitive disorders

**Figure 4:**
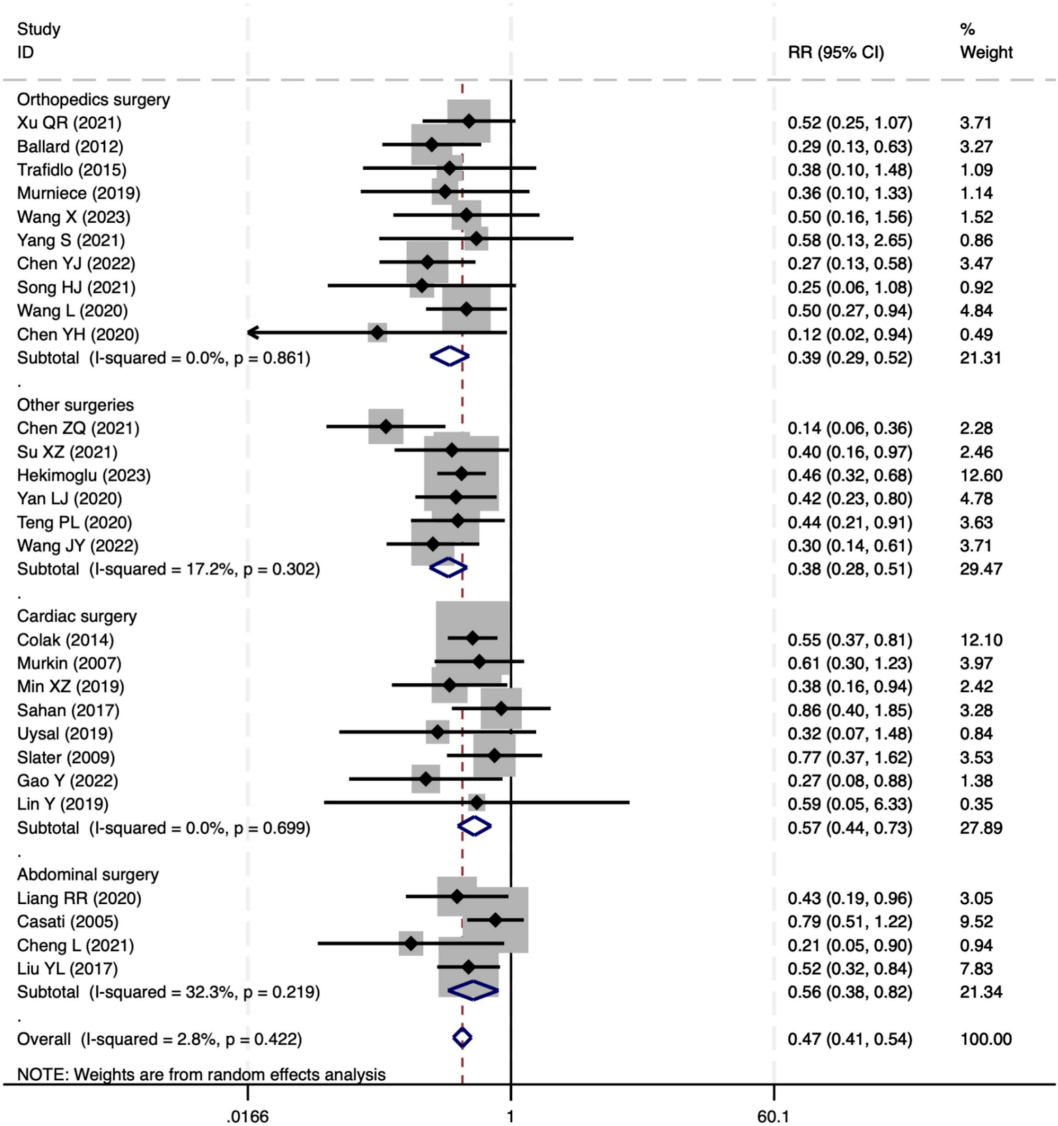
The forest plot of the impact of cerebral oxygen saturation on PND.

**Figure 5:**
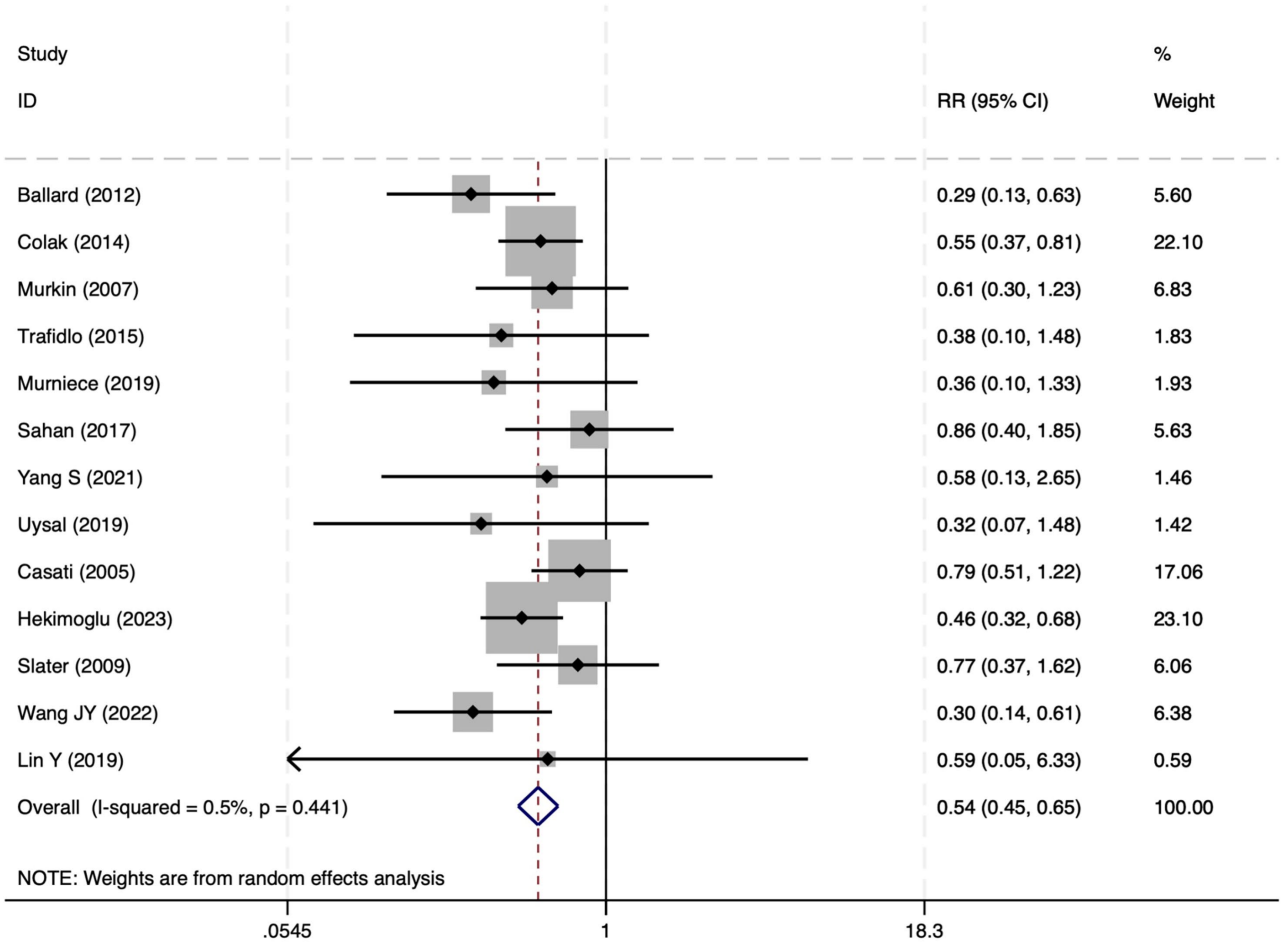
The forest plot of the impact of cerebral oxygen saturation on PND after excluding Chinese-language studies.

**Figure 6:**
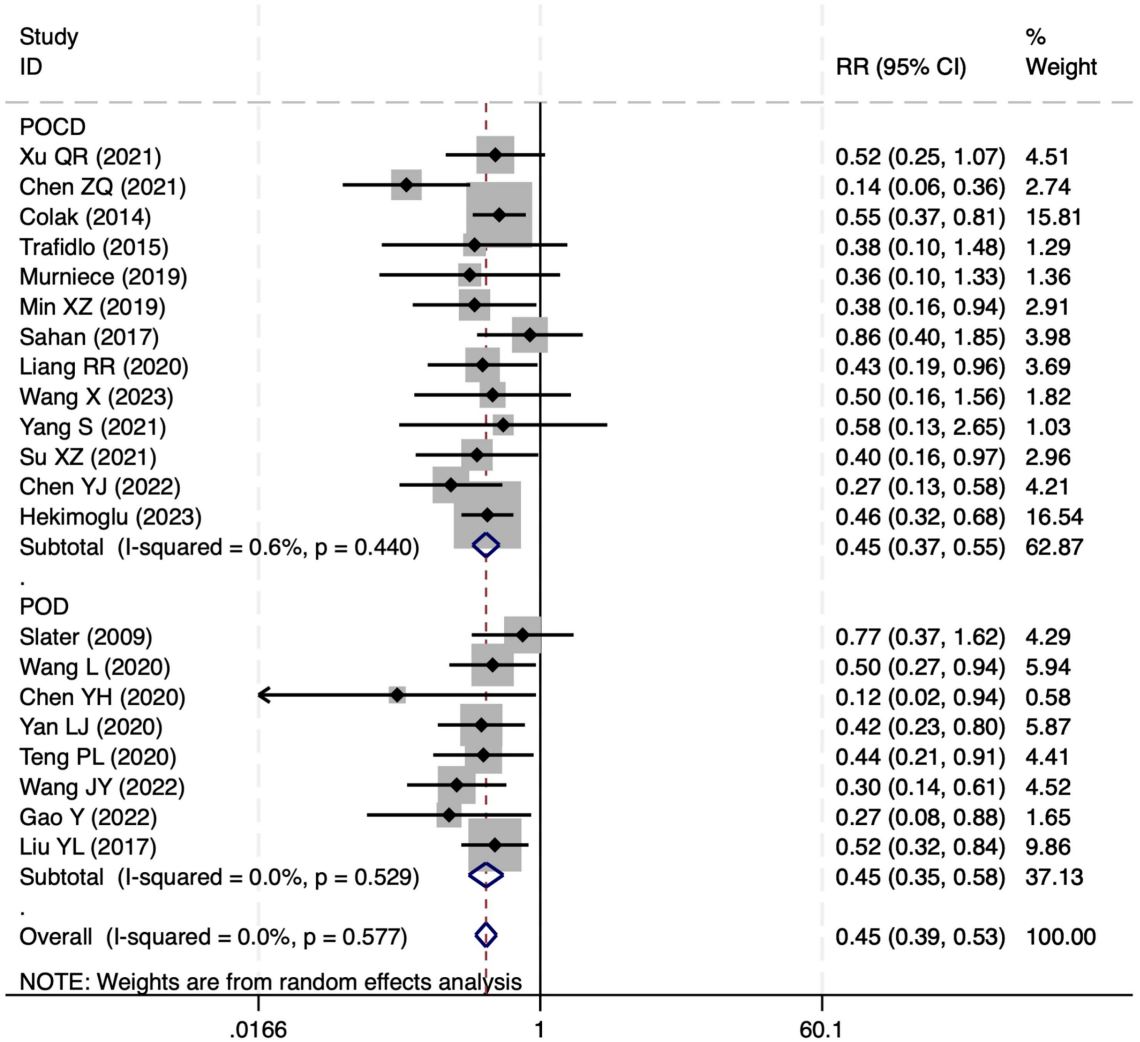
Forest plot after excluding studies where rScO_2_ monitoring was applied only when the baseline was below 80%.

**Figure 7:**
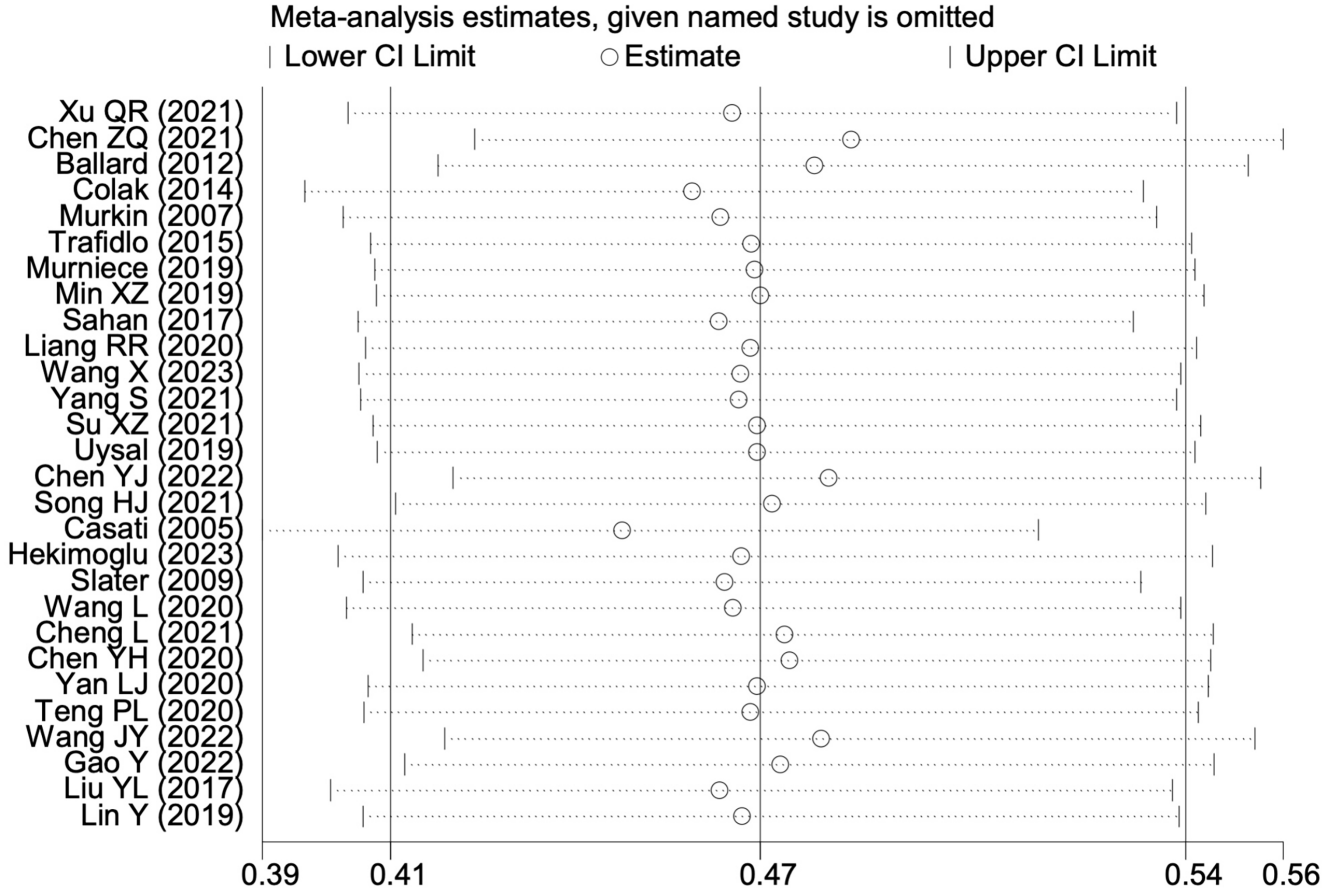
Sensitivity analysis of the effect of intraoperative cerebral oximetry monitoring on the incidence of PND using the leave-one-out method.

**Figure 8:**
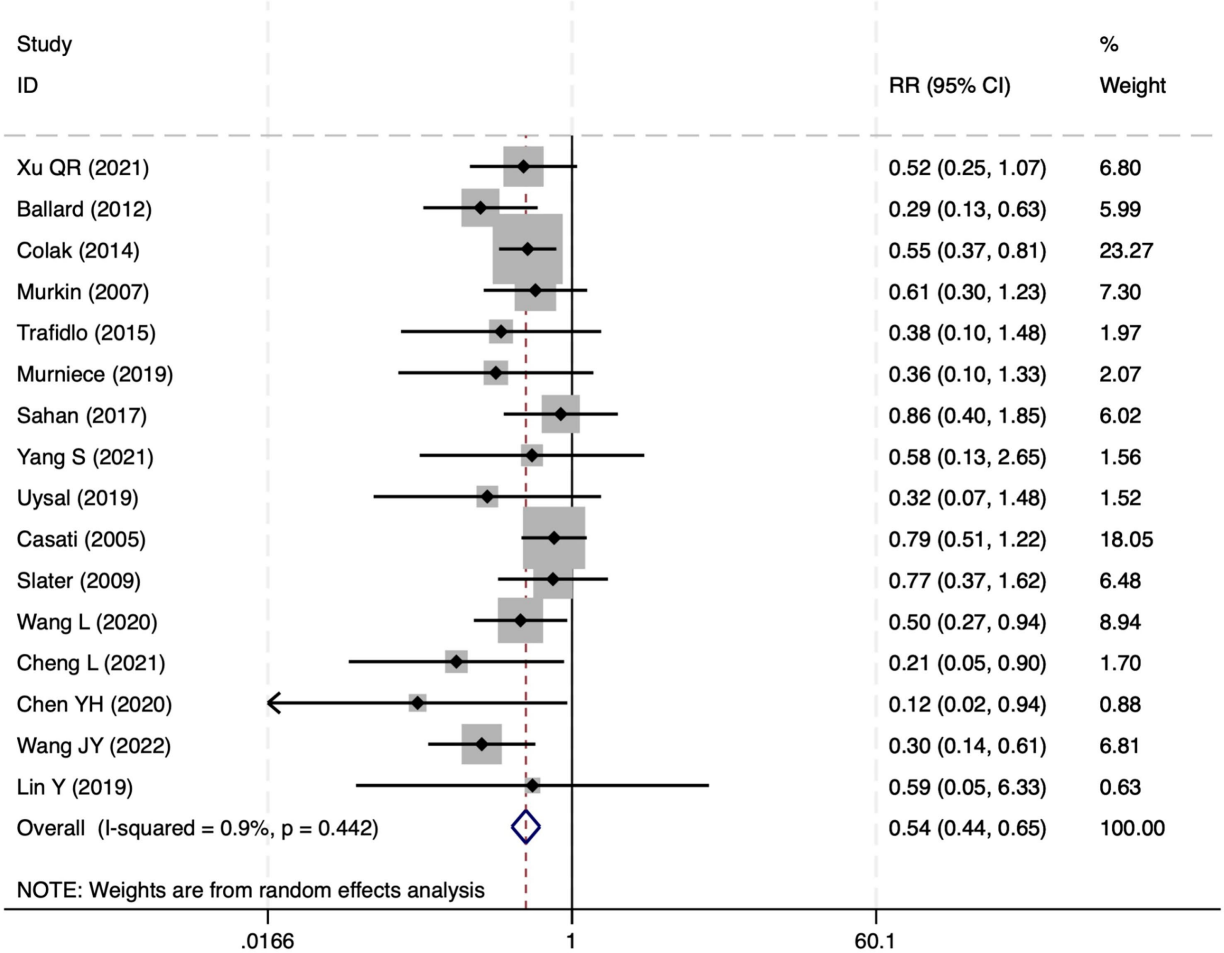
The forest plot of the impact of cerebral oxygen saturation on PND after excluding low-quality studies.

**Figure 9:**
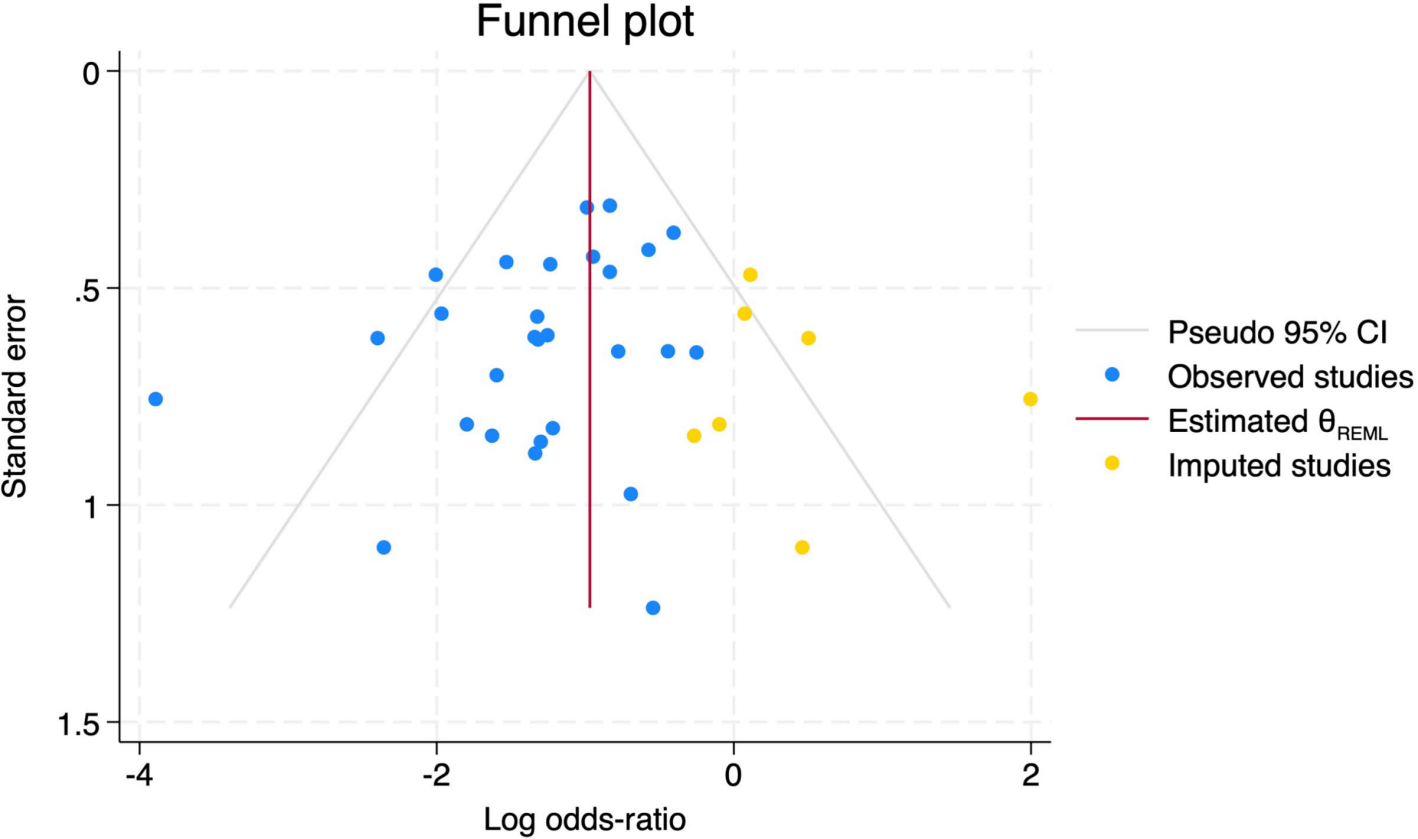
Trim and fill method for PND.

All 28 included studies reported PNDs. Overall, the experimental group that underwent intraoperative rScO_2_ monitoring had a significantly lower incidence risk of PND (RR = 0.47, 95% CI: 0.41, 0.54; *I*^2^ = 2.8%, *P*_h_ = 0.422; Figure 4) than the control group without monitoring. We then conducted subgroup analyses according to the type of surgery, which included cardiac surgery, orthopedic surgery, abdominal surgery, and other types of surgery. We found that the following subgroups of the experimental group that underwent rScO_2_ monitoring had a significantly reduced risk of postoperative PND: cardiac surgery group (RR = 0.57, 95% CI: 0.44, 0.73; *I^2^* = 0.0%, *P_h_* = 0.699); orthopedic surgery group (RR = 0.39, 95% CI: 0.29, 0.52; *I^2^* = 0.0%, *P_h_* = 0.861); abdominal surgery group (RR = 0.56, 95% CI: 0.38, 0.82; *I^2^* = 32.3%, *P_h_* = 0.219); other surgery group (RR = 0.38, 95% CI: 0.28, 0.51; *I^2^* = 17.2%, *P_h_* = 0.302). Other subgroup analyses were presented in Supplementary Table 2. A significant portion of the included studies originated from China, which may introduce geographic and population bias. To address this concern, we conducted a subgroup analysis excluding all Chinese-language articles. The results showed that compared with the control group without intraoperative rScO_2_ monitoring, the experimental group exhibited a significantly lower risk of PND (RR = 0.54, 95% CI: 0.45, 0.65; *I^2^* = 0.5%, *P_h_* = 0.441; Figure 5). The stability of these findings suggested that our results can be generalized to broader and more diverse patient populations. After excluding studies in which intraoperative interventions were applied only when rScO_2_ dropped below 80% of baseline, the risk of PND in the experimental group remained significantly lower than that in the control group without intraoperative rScO_2_ monitoring (RR = 0.45, 95% CI: 0.39, 0.53; *I^2^* = 0.0%, *P_h_* = 0.577; Figure 6). The sensitivity analysis using the one-by-one elimination method revealed no changes (Figure 7). Moreover, when 12 low-quality studies (22, 23, 25, 26, 34–37, 40, 41, 43, 45) were excluded, there was also no change in the results (RR = 0.54, 95% CI: 0.44, 0.65; *I^2^* = 0.9%, *P_h_* = 0.442; Figure 8), which further indicated that the pooled results were robust and reliable. The distribution of the funnel plot and the result of Egger’s (*p* = 0.012) and Begg’s tests (*p* = 0.023) indicated the presence of publication bias. Using the trim-and-fill method for adjustment (Figure 9), the pooled effect remained robust (RR = 0.38, 95% CI: 0.28, 0.51) without change in the direction of the result. The GRADE assessment for PND is shown in Supplementary Figure 1.

#### Postoperative cognitive dysfunction

**Figure 10:**
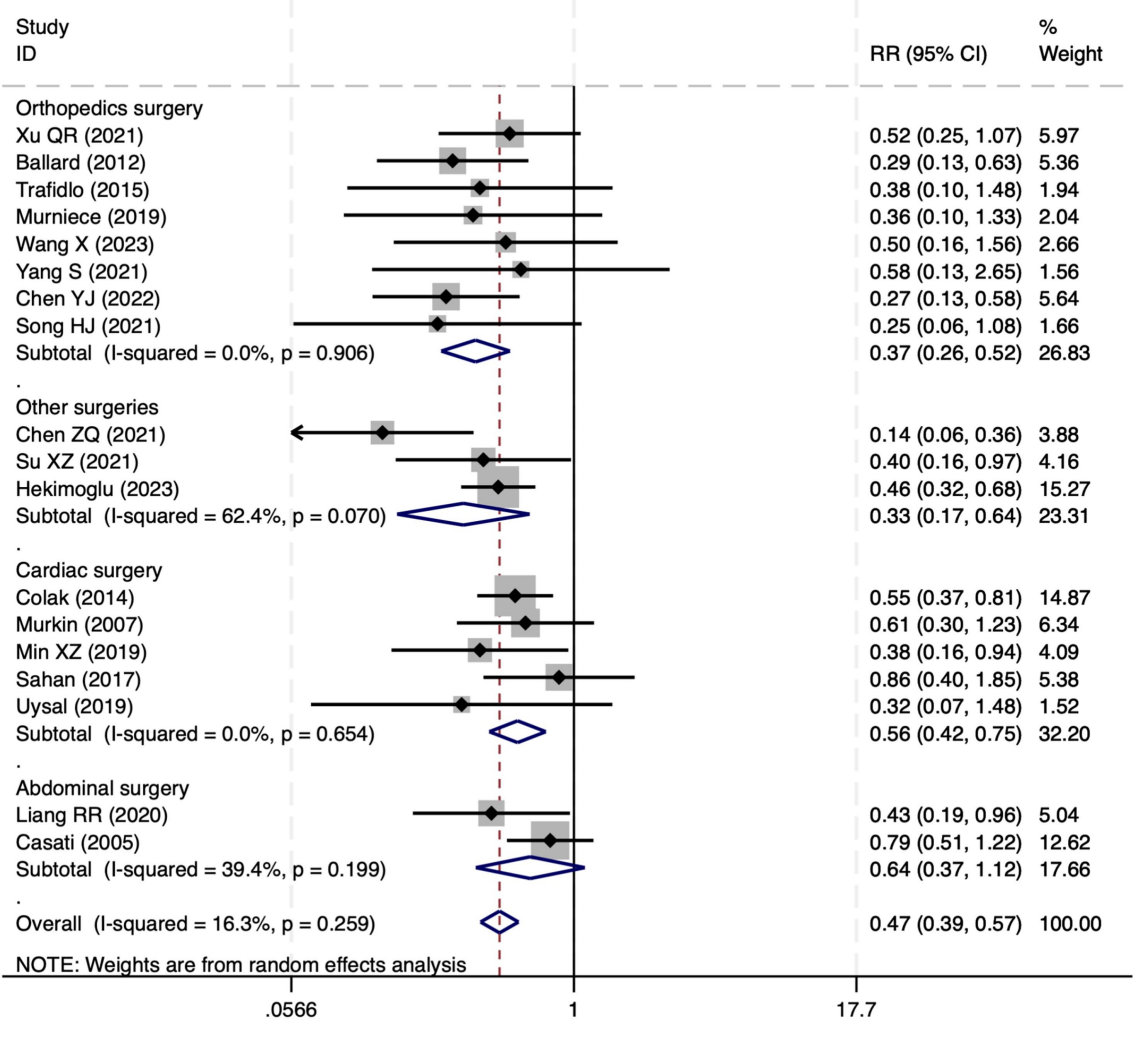
The forest plot of the impact of cerebral oxygen saturation on POCD.

**Figure 11:**
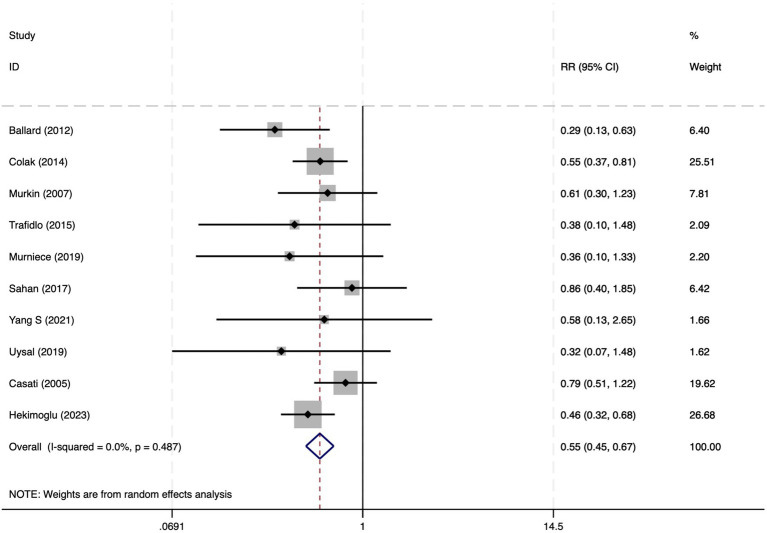
The forest plot of the impact of cerebral oxygen saturation on POCD after excluding Chinese-language studies.

**Figure 12:**
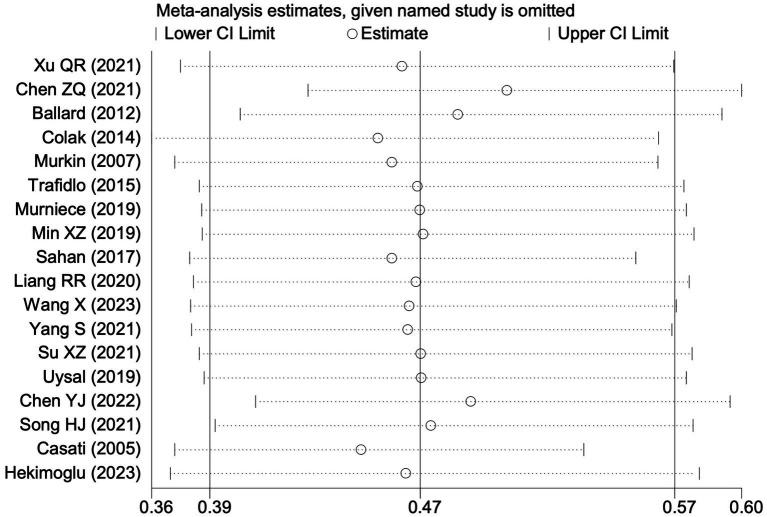
Sensitivity analysis of the effect of intraoperative cerebral oximetry monitoring on the incidence of POCD using the leave-one-out method.

**Figure 13:**
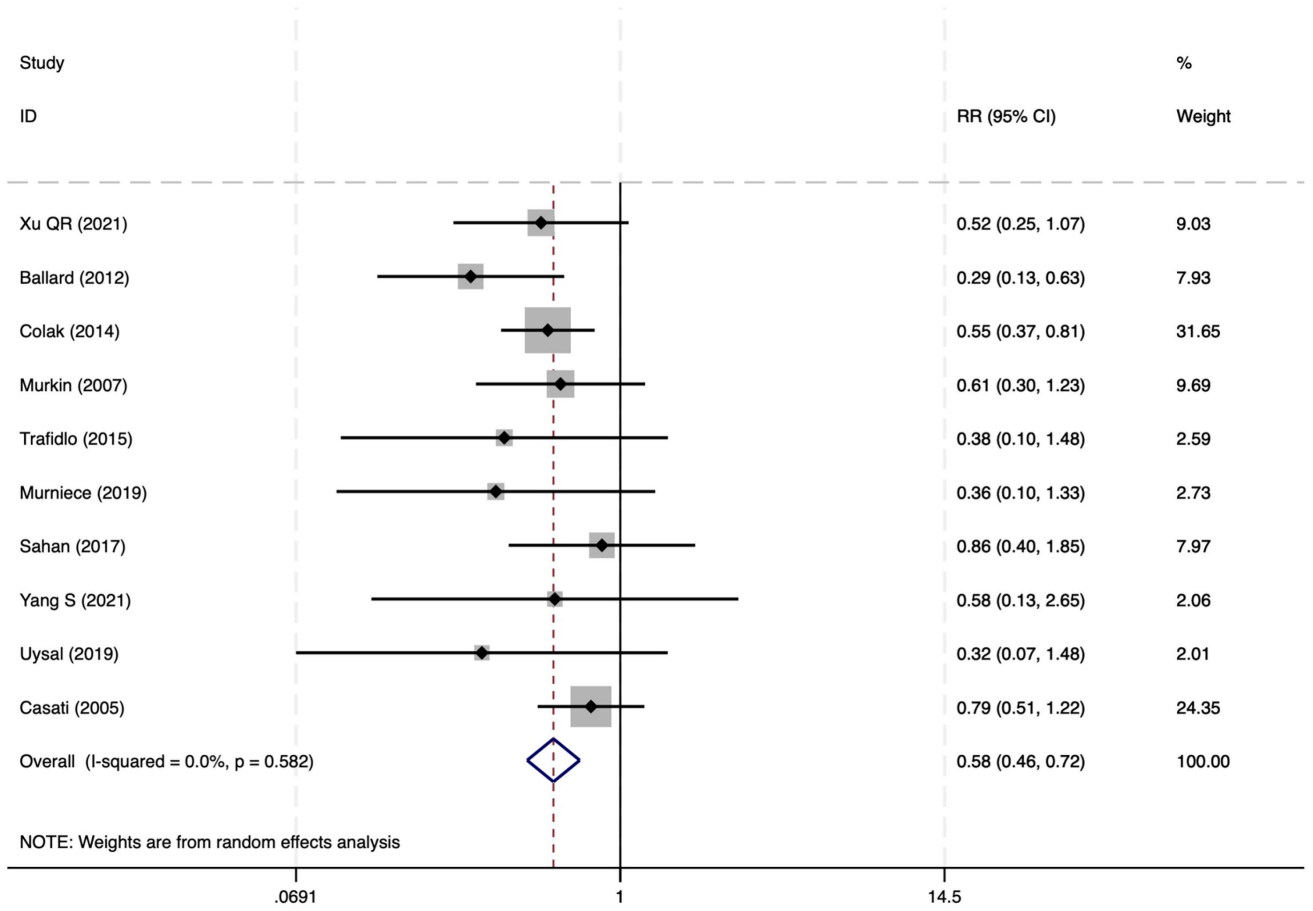
The forest plot of the impact of cerebral oxygen saturation on POCD after excluding low-quality studies.

**Figure 14:**
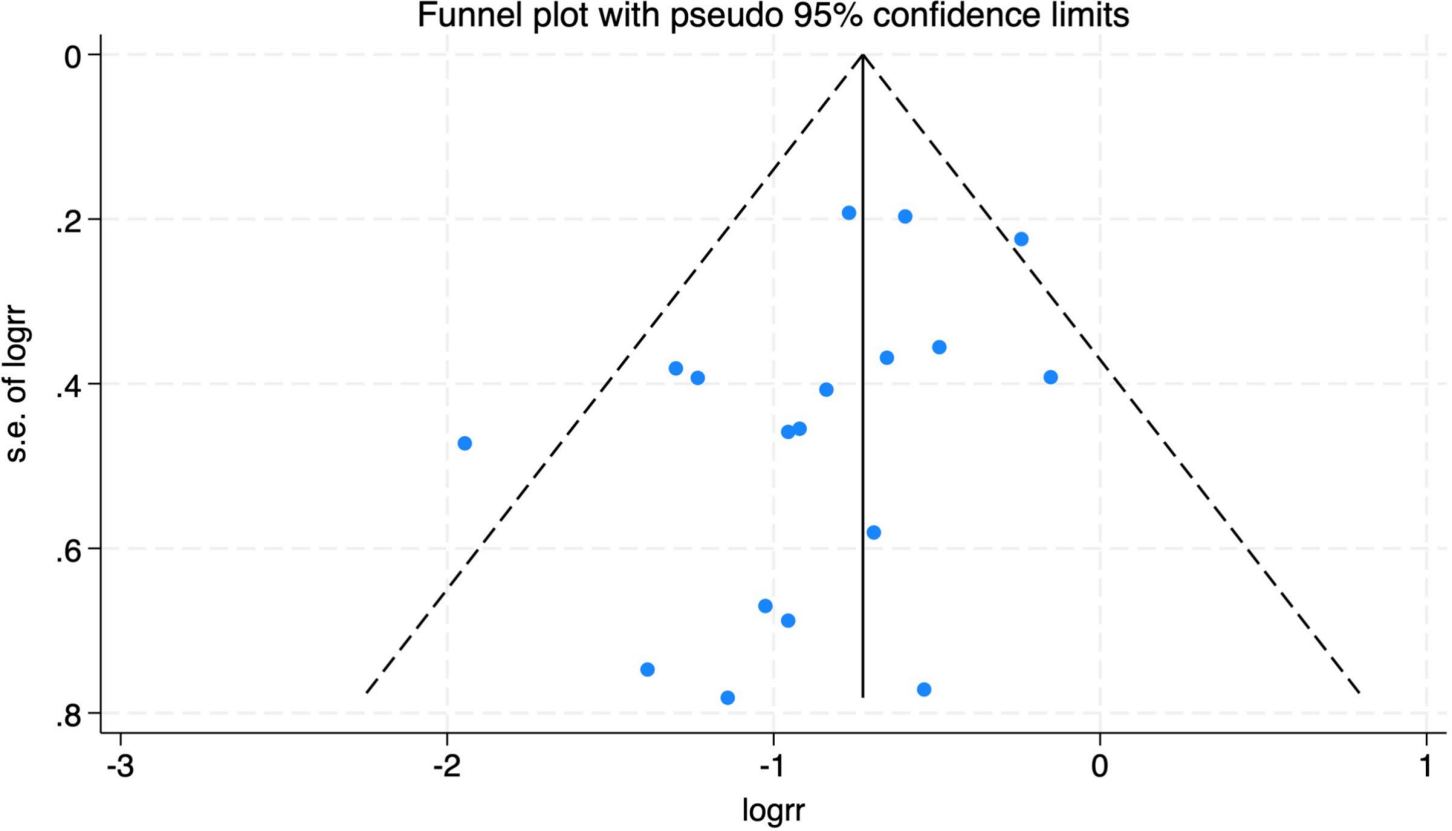
The funnel plot of the impact of cerebral oxygen saturation on POCD.

Among the 28 included studies, 18 reported on POCD. Overall, a lower incidence risk of POCD was observed (RR = 0.47, 95% CI: 0.39, 0.57; *I*^2^ = 16.3%, *P*_h_ = 0.259; Figure 10) in the intervention group that underwent intraoperative rScO_2_ monitoring than in the control group that did not undergo monitoring. The subgroup analysis results by surgery type showed that rScO2 monitoring tended to reduce POCD risk across surgery types, especially in cardiac and orthopedic surgeries, where the effect is more significant and consistent cardiac surgery group (RR = 0.56, 95% CI: 0.42, 0.75; *I^2^* = 0.0%, *P_h_* = 0.654); orthopedic surgery group (RR = 0.37, 95% CI: 0.26, 0.52; *I^2^* = 0.0%, *P_h_* = 0.906); abdominal surgery group (RR = 0.64, 95% CI: 0.37, 1.12; *I^2^* = 39.4%, *P_h_* = 0.199); other surgery group (RR = 0.33, 95% CI: 0.17, 0.64; *I^2^* = 62.4%, *P_h_* = 0.070). Other subgroup analyses were presented in Supplementary Table 2. The subgroup analysis excluding all Chinese-language articles showed that compared with the control group without intraoperative rScO_2_ monitoring, the experimental group exhibited a significantly lower risk of POCD (RR = 0.55, 95% CI: 0.45, 0.67; *I^2^* = 0.0%, *P_h_* = 0.487; Figure 11). After excluding studies in which intraoperative interventions were applied only when rScO_2_ dropped below 80% of baseline, the risk of POCD in the experimental group remained significantly lower than that in the control group without intraoperative rScO_2_ monitoring (RR = 0.45, 95% CI: 0.37, 0.55; *I^2^* = 0.6%, *P_h_* = 0.440; Figure 6). The sensitivity analysis did not change the above results (Figure 12). Additionally, when eight low-quality studies (25, 26, 34, 36, 37, 40, 43, 45) were excluded, the results did not change (RR = 0.58, 95% CI: 0.46, 0.72; *I^2^* = 0.0%, *P_h_* = 0.582; Figure 13), which indicated that the pooled results were robust and reliable. Although the Egger’s (*p* = 0.070) and Begg’s tests (*p* = 0.081) did not indicate significant publication bias, the funnel plot (Figure 14) showed a slight asymmetry. Therefore, we performed a trim-and-fill analysis. The analysis did not impute any missing studies, which suggested minimal risk of publication bias and confirmed the robustness of the pooled results. The GRADE assessment for POCD is shown in Supplementary Figure 1.

#### Postoperative delirium

**Figure 15:**
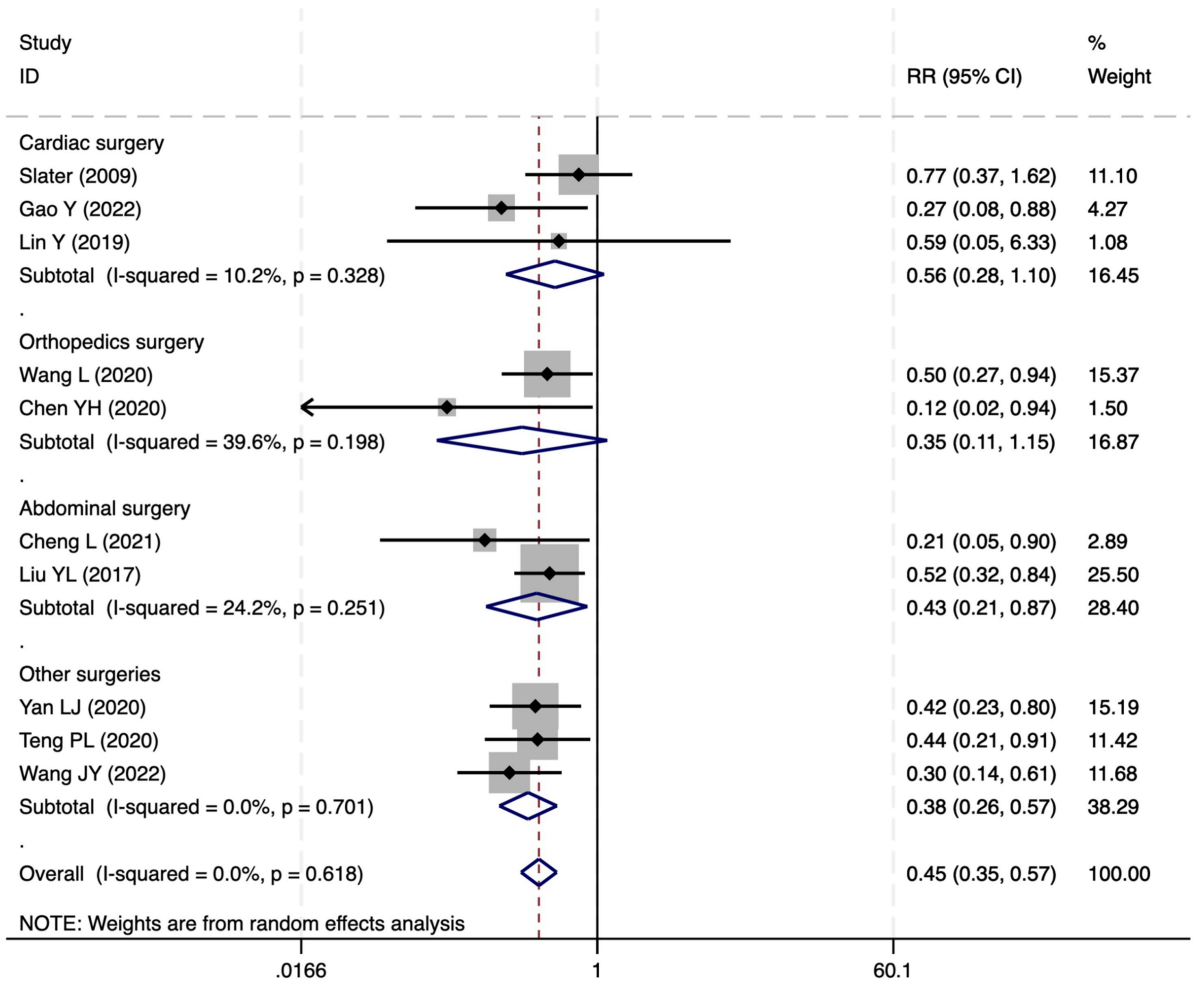
The forest plot of the impact of cerebral oxygen saturation on POD.

**Figure 16:**
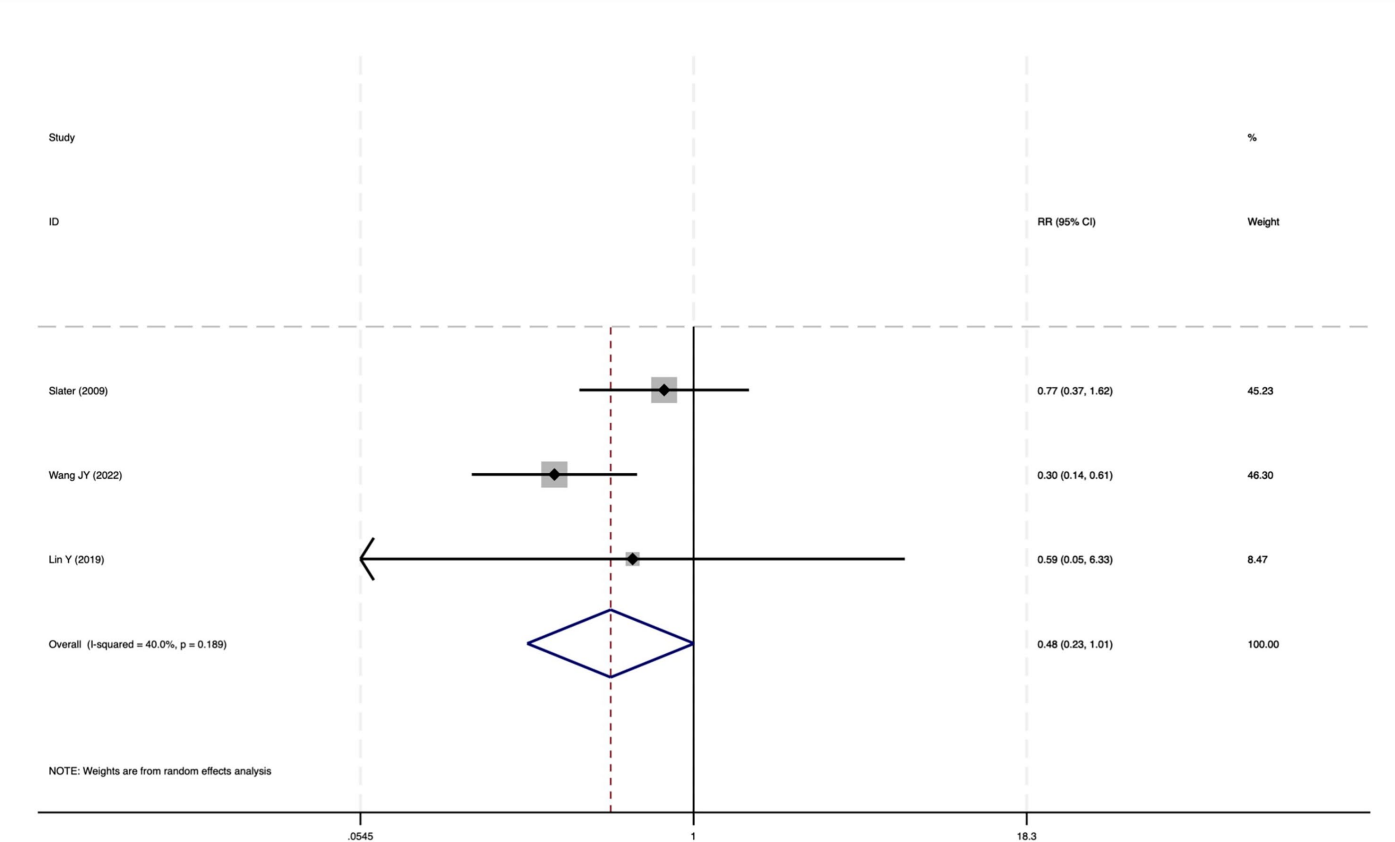
The forest plot of the impact of cerebral oxygen saturation on POD after excluding Chinese-language studies.

**Figure 17:**
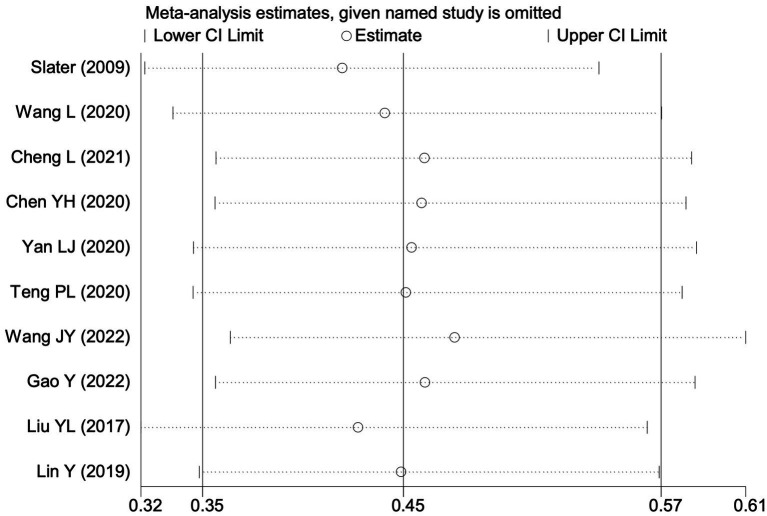
Sensitivity analysis of the effect of intraoperative cerebral oximetry monitoring on the incidence of POD using the leave-one-out method.

**Figure 18:**
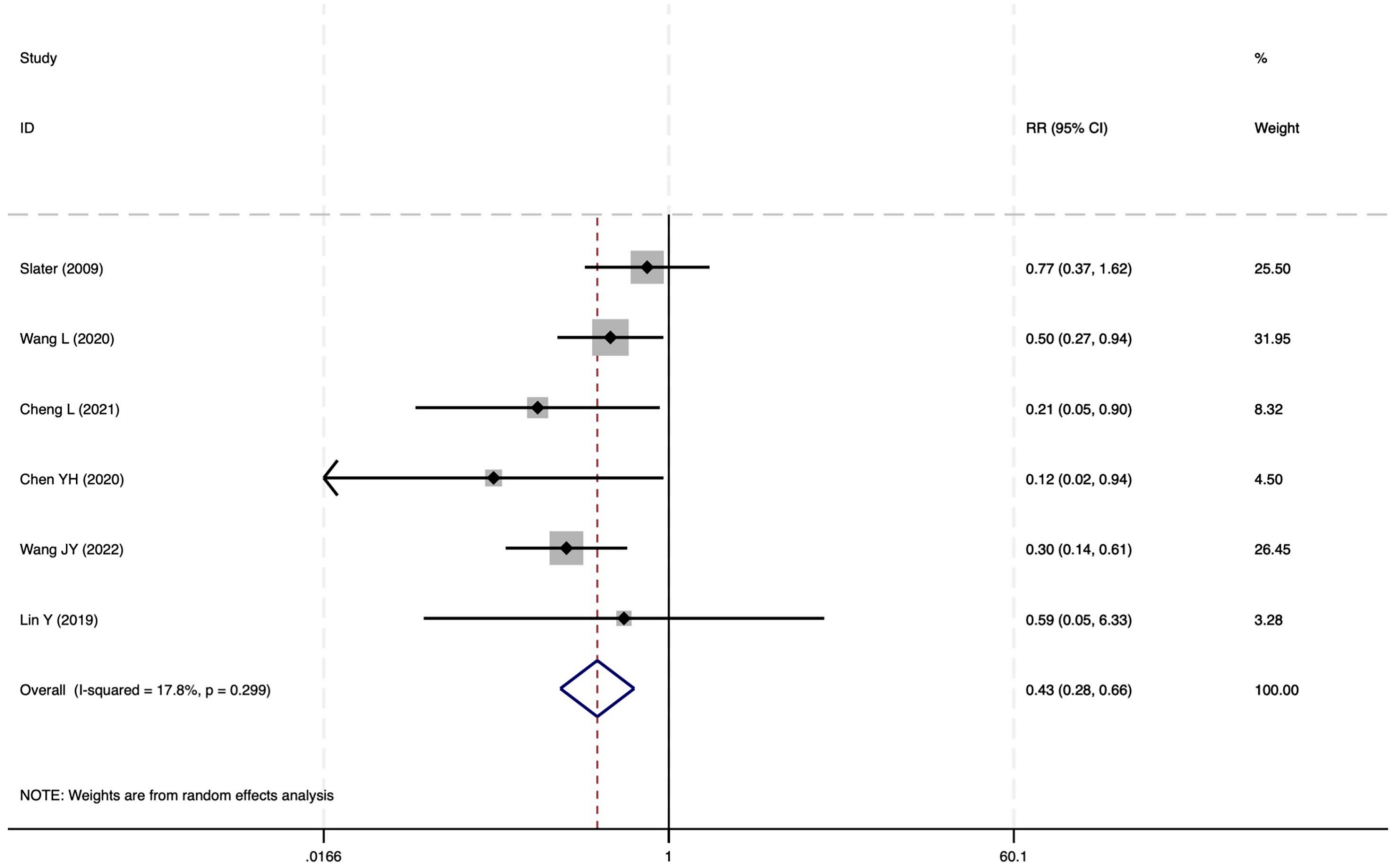
The forest plot of the impact of cerebral oxygen saturation on POD after excluding low-quality studies.

**Figure 19:**
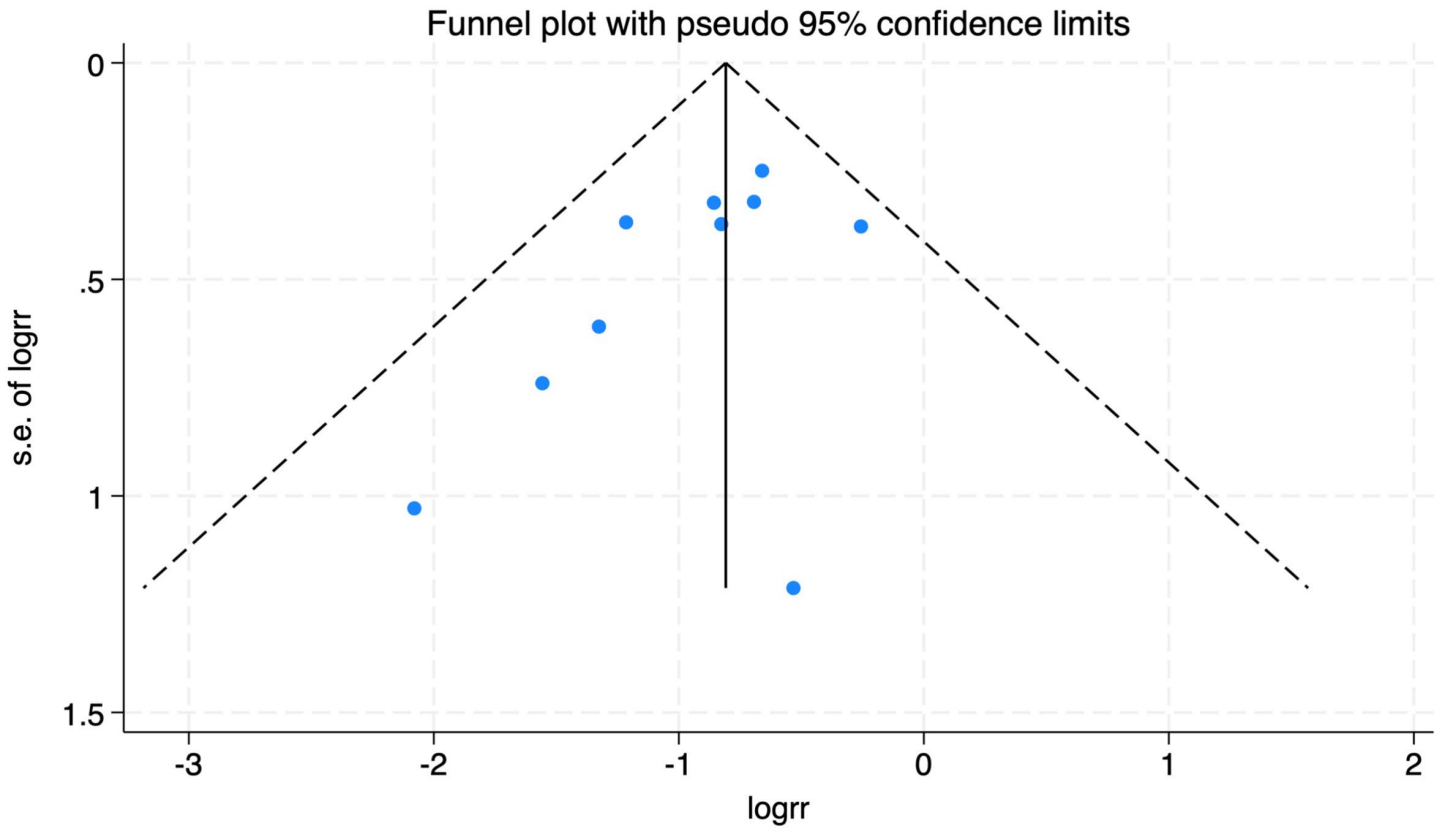
The funnel plot of the impact of cerebral oxygen saturation on POD.

**Figure 20:**
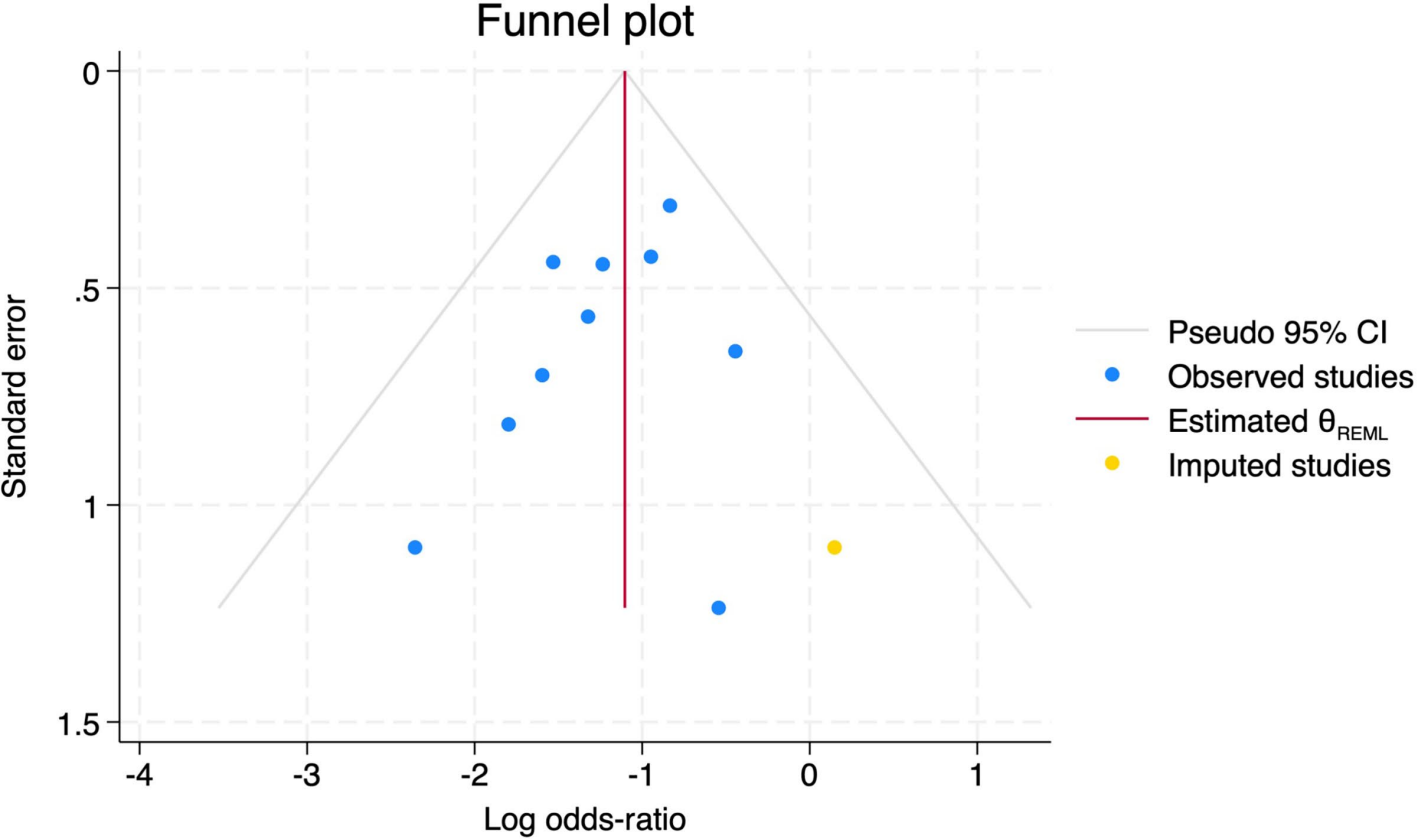
Trim and fill method for POD.

Of the 28 included studies, 10 reported on POD. Overall, a lower incidence risk of POD was observed (RR = 0.45, 95% CI: 0.35, 0.57; *I*^2^ = 0.0%, *P*_h_ = 0.618; Figure 15) in the intervention group that underwent intraoperative rScO_2_ monitoring than in the control group that did not undergo monitoring. In addition, there was a trend of monitoring reducing the risk of POD across different surgery types, particularly abdominal (RR = 0.43, 95% CI: 0.21, 0.87; *I^2^* = 24.2%, *P_h_* = 0.251; Figure 15) and other surgeries (RR = 0.38, 95% CI: 0.26, 0.57; *I^2^* = 0.0%, *P_h_* = 0.701; Figure 15), which were significant. Other subgroup analyses were presented in Supplementary Table 2. When all Chinese-language articles were excluded, only three studies remained. When we compared the control and monitoring groups, intraoperative rScO_2_ monitoring showed a trend toward a reduced risk of POD (RR = 0.48, 95% CI: 0.23, 1.01; *I^2^* = 40.0%, *P_h_* = 0.189; Figure 16), although this did not reach significance. After excluding studies in which intraoperative interventions were applied only when rScO_2_ dropped below 80% of baseline, the risk of POD in the experimental group remained significantly lower than that in the control group without intraoperative rScO_2_ monitoring (RR = 0.45, 95% CI: 0.35, 0.58; *I^2^* = 0.0%, *P_h_* = 0.529; Figure 6). The sensitivity analysis did not change the above results (Figure 17). Moreover, when four low-quality studies (22, 23, 35, 41) were excluded, the results did not change (RR = 0.43, 95% CI: 0.28, 0.66; *I^2^* = 17.8%, *P_h_* = 0.299; Figure 18), which further indicated that the pooled results were robust and reliable. Although the Egger’s (*p* = 0.121) and Begg’s tests (*p* = 0.210) did not indicate significant publication bias, the funnel plot (Figure 19) showed a slight asymmetry. Therefore, we performed a trim-and-fill analysis (Figure 20). The pooled effect remained robust (RR = 0.33, 95% CI: 0.24, 0.46) without no change in the direction of the result. The GRADE assessment for POD is shown in Supplementary Figure 1.

#### Economic evaluation of cerebral oximetry in reducing postoperative neurological complications

The calculated effect indicators (Table 2) of ARR, NNT, and CBR collectively demonstrated that cerebral oximetry was associated with a substantial reduction in the risk of PND, POCD, and POD. The NNT to prevent one case of PND was approximately 5.0 (95% CI: 4.2, 6.0), which suggested that monitoring five patients could prevent one additional case. Similarly, the NNTs for POCD and POD were approximately 4.5 (95% CI: 3.7, 5.6) and 5.7 (95% CI: 4.4, 8.1), respectively. Such low NNT values indicated that the intervention is clinically meaningful because a substantial number of adverse outcomes can be prevented by offering rScO_2_ monitoring to a relatively small number of patients.

**Table 2:** Clinical and economic benefits of cerebral oxygen saturation monitoring for preventing postoperative neurocognitive disorders.

Outcome	Subgroup	Experimental group			Control group			RR (95% CI)	NNT (95% CI)	ARR (%) (95% CI)	CBR (95% CI)
		Total (n1)	Events (a1)	Risk (p1)	Total (n2)	Events (a2)	Risk (p2%)				
PND	Overall	1,295	209	16.1%	1,329	483	36.3%	0.47 (0.41 ~ 0.54)	5.0 (4.2 ~ 6.0)	20.2 (16.7 ~ 23.7)	0.058 (0.047 ~ 0.070)
	Cardiac	416	61	14.7%	442	118	26.7%	0.57 (0.44 ~ 0.73)	8.3 (5.6 ~ 15.9)	12.0 (6.3 ~ 17.7)	0.096 (0.066 ~ 0.188)
	Orthopedic	344	46	13.4%	354	128	36.2%	0.39 (0.29 ~ 0.52)	4.4 (3.5 ~ 6.0)	22.8 (16.7 ~ 28.9)	0.051 (0.041 ~ 0.063)
	Abdominal	264	49	18.6%	257	87	33.9%	0.56 (0.38 ~ 0.82)	6.5 (4.2 ~ 14.7)	15.3 (6.8 ~ 23.8)	0.075 (0.049 ~ 0.153)
	Other	271	53	19.6%	276	150	54.3%	0.38 (0.28 ~ 0.51)	2.9 (2.3 ~ 3.8)	34.7 (26.3 ~ 43.1)	0.034 (0.027 ~ 0.045)
POCD	Overall	750	139	18.5%	772	315	40.8%	0.47 (0.39 ~ 0.57)	4.5 (3.7 ~ 5.6)	22.3 (17.9 ~ 26.7)	0.052 (0.042 ~ 0.064)
	Cardiac	296	50	16.9%	310	95	30.6%	0.56 (0.42 ~ 0.75)	7.3 (4.9 ~ 14.5)	13.7 (6.9 ~ 20.5)	0.085 (0.058 ~ 0.166)
	Orthopedic	254	34	13.4%	264	98	37.1%	0.37 (0.26 ~ 0.52)	4.2 (3.2 ~ 6.1)	23.7 (16.4 ~ 31.0)	0.049 (0.037 ~ 0.072)
	Abdominal	90	27	30%	87	40	46%	0.64 (0.37 ~ 1.12)	6.3 (2.9 ~ ∞)	16.0 (−3.0 ~ 35.0)	0.073 (0.034 ~ ∞)
	Other	110	28	25.5%	111	82	73.9%	0.33 (0.17 ~ 0.64)	2.1 (1.5 ~ 3.1)	48.4 (32.1 ~ 64.7)	0.024 (0.017 ~ 0.036)
POD	Overall	545	70	12.8%	557	168	30.2%	0.45 (0.35 ~ 0.57)	5.7 (4.4 ~ 8.1)	17.4 (12.3 ~ 22.5)	0.066 (0.051 ~ 0.092)
	Cardiac	120	11	9.2%	132	23	17.4%	0.56 (0.28 ~ 1.10)	12.2 (5.3 ~ ∞)	8.2 (−2.3 ~ 18.7)	0.141 (0.062 ~ ∞)
	Orthopedic	90	12	13.3%	90	30	33.3%	0.35 (0.11 ~ 1.15)	5.0 (2.7 ~ 30.3)	20.0 (3.3 ~ 36.7)	0.058 (0.032 ~ 0.585)
	Abdominal	174	22	12.6%	170	47	27.6%	0.43 (0.21 ~ 0.87)	6.7 (3.9 ~ 23.8)	15.0 (4.2 ~ 25.8)	0.078 (0.047 ~ 0.231)
	Other	161	25	15.5%	165	68	41.2%	0.38 (0.26 ~ 0.57)	3.9 (2.8 ~ 6.2)	25.7 (16.1 ~ 35.3)	0.045 (0.033 ~ 0.060)

NNT, number needed to treat; ARR, attributable risk; CBR, Cost–Benefit Ratio, calculated as intervention cost per benefit unit; CI, confidence interval; PND, perioperative neurocognitive disorders; POCD, postoperative cognitive dysfunction; POD, postoperative delirium.

From an economic perspective, the CBR analysis was based on estimates reported in the literature, which suggest that cerebral oximetry costs approximately $200 per patient (46), whereas the occurrence of postoperative neurological complications (including PND, POCD, and POD) would result in an additional average cost of approximately $17,275 per case (47). This cost differential was applied uniformly across all neurological outcomes to enable a standardized economic evaluation. The intervention cost of rScO_2_ monitoring to prevent one case of PND is approximately $1,000 (5 × $200), which is markedly lower than the associated financial burden of postoperative neurological complications. These findings, together with favorable CBR values, suggest that cerebral oximetry is not only clinically beneficial but also cost-effective, supporting its broader adoption in perioperative care.

Overall, the RR values ranged from 0.43 to 0.47, which corresponds to a relative risk reduction of over 50%. The ARR exceeded 17% for all outcomes, with NNTs falling predominantly between 4 and 6, indicating strong clinical impact. Subgroup analyses revealed particularly pronounced effects for orthopedic and miscellaneous surgeries, which showed higher ARRs and lower NNTs, although the benefit was relatively modest for cardiac surgeries. The CBR values further underscore the potential of cerebral oximetry for broader perioperative implementation, especially during procedures involving a higher baseline risk of neurocognitive impairment.

## Discussion

We conducted a meta-analysis of 28 RCTs to systematically evaluate the impact of intraoperative rScO_2_ monitoring on the incidence of PND, POCD, and POD. The results indicated that the use of rScO_2_ monitoring and corresponding interventions during surgery significantly reduced the incidence of PND, POCD, and POD across various surgery types. Our findings align with previous studies, underscoring the importance of rScO_2_ monitoring in perioperative management, and provide strong evidence for broader clinical implementation of this monitoring strategy (48) to offer additional brain protection and a solid foundation for improving patients’ long-term quality of life (49).

In addition to the clinical benefits, rScO_2_ monitoring demonstrates good economic value. Based on the NNT values for PND, POCD, and POD (ranging from 4.5 to 5.7) and an estimated cost of $200 per patient, the total cost of preventing a single neurocognitive event was significantly lower than the average economic burden of such complications, which can reach $17,275 per case. Therefore, monitoring rScO_2_ is not only effective but also cost-effective, especially in those undergoing orthopedic surgery. Walsh et al. (50) conducted a cost analysis on 100 patients undergoing cardiac surgery and compared those who underwent cerebral oxygen monitoring (via the INVOS system) with those without monitoring. They found that although the INVOS device itself came with a cost, the patient group that used INVOS significantly reduced their postoperative hospital stay (including in both the intensive care unit and the general wards), thereby enabling a considerable cost saving. Overall, approximately £102,000 could be saved for every 100 patients who undergo heart surgery, which indicates that rScO_2_ monitoring not only significantly reduces the risk of postoperative neurological complications but also offers significant economic benefits by shortening the length of hospital stay.

In non-cardiac surgeries, such as orthopedic and abdominal surgeries, the preventive effect of rScO_2_ monitoring on POCD and POD was particularly pronounced. The protective effect of rSO_2_ monitoring on preventing POCD is more obvious in non-cardiac surgeries. This might be because global cerebral ischemia and hypoxia caused by systemic hypotension or anemia in non-cardiac surgeries are more easily reflected through rSO_2_ monitoring, while the distribution of cerebral ischemia in cardiac surgeries may be uneven, resulting in a less significant effect of rSO_2_ monitoring compared to non-cardiac surgeries (51). This may be because patients undergoing non-cardiac surgeries experience less physiological fluctuation than those undergoing cardiac surgeries; thus, rScO_2_ monitoring allows more efficient response to localized cerebral hypoxia. By contrast, during cardiac surgeries, where patients experience more complex and dramatic circulatory changes and greater instability in cerebral blood flow and oxygen supply (52), rScO_2_ monitoring offers critical protection, which, in turn, significantly reduces the incidence of PND. This demonstrates the clinical value of rScO_2_ monitoring across different types of surgeries and further validates its wide applicability as an intraoperative monitoring tool. Therefore, we recommend that rScO_2_ monitoring be implemented broadly in various surgeries to improve patients’ postoperative cognitive outcomes.

Notably, the studies included in this meta-analysis encompassed various types of surgeries, including cardiac procedures (e.g., coronary artery bypass grafting and valve replacement) and non-cardiac surgeries (e.g., orthopedic and abdominal surgeries). The interventions were based on NIRS monitoring of rScO_2_ and included personalized management strategies, such as adjustments to inspired oxygen concentration, circulatory management, and other measures aimed at optimizing cerebral oxygen supply (53). Such diversity in procedures highlights the robustness of the observed intervention effects and suggests that the benefits of rScO_2_ monitoring vary depending on the type of surgery and patient population.

Despite these findings, this meta-analysis has several limitations. First, the number of included studies was limited, which may contribute to statistical heterogeneity and affect the robustness of the findings. Second, patient-reported outcomes and functional measures, such as quality of life, were not assessed. Therefore, the comprehensiveness of postoperative recovery evaluation was limited. Third, the lack of standardized thresholds for rScO_2_ monitoring across studies restricts the reproducibility and clinical applicability of the results. Fourth, only two studies reported outcomes at 3 months, limiting the strength of conclusions regarding long-term effects. Fifth, about 60% of patients were recruited in Chinese centers; although excluding Chinese-language studies did not change the PND result, the number of non-Chinese trials was small for some outcomes (e.g., POD), so broad generalisability should be cautioned and multicentre data from other regions are needed. Finally, we did not stratify PND, POCD, or POD by severity or duration, which may limit the clinical relevance of the findings. Until consensus definitions exist, we recommend that future trials adopt CAM/CAM-ICU for POD and the ISPOCD 1-SD rule for POCD assessed at 1 week and 3 months to facilitate comparison and clinical translation. Supplementary Table 3 shows that most studies used the same three-step bundle (raise FiO_2_, MAP and Hb), sensitivity analyses found similar RRs after excluding trials that waited until rScO_2_ < 80% baseline, suggesting the benefit comes from the bundle, not the trigger value. Future research should address these issues via standardized protocols, longer follow-up periods, and comprehensive neurocognitive outcome measures.

Overall, intraoperative rScO_2_ monitoring is an effective means of preventing PNDs in patients undergoing general anesthesia. Although our findings are encouraging, larger, multicenter RCTs are needed to validate our results further. Given the significant results of rScO_2_ monitoring and management, further investigation into its specific mechanisms and clinical applications will provide anesthesiologists with more precise guidance on the management of general anesthesia surgeries. The traditional focus of anesthesia management has seen a shift from merely ensuring stable vital signs to also considering the potential impact of anesthesia on the brain. By implementing rScO_2_ monitoring, not only is patient safety during surgery ensured but also cognitive health is safeguarded.

## Conclusion

This study revealed that rScO_2_ monitoring, as an intraoperative intervention, significantly reduces the occurrence of PND. Moreover, it has wide clinical applicability across all types of surgeries. Its widespread implementation is recommended to improve the postoperative cognitive outcomes of patients undergoing surgery, especially given its cost-effectiveness in reducing postoperative neurocognitive complications.

### What is known?

1. Inadequate cerebral oxygen supply during anesthesia and surgery is a risk factor for perioperative neurocognitive disorders (PNDs).
2. Although previous studies have explored the impact of intraoperative rScO_2_ monitoring on cognitive function, results have been inconsistent; moreover, outcomes were not classified by surgery type.

### What is new?

1. This meta-analysis demonstrated that intraoperative rScO_2_ monitoring significantly reduces the incidence of PND, POCD, and POD.
2. RScO_2_ monitoring is beneficial for both cardiac and non-cardiac surgeries, showing its broad applicability across different patient populations.
3. Our findings support the widespread implementation of rScO_2_ monitoring as an effective intervention to improve postoperative cognitive outcomes.
4. Our analysis revealed favorable economic benefits with a low CBR, which indicates that rScO_2_ monitoring is a cost-effective strategy for preventing postoperative neurocognitive complications.

## Data Availability

The original contributions presented in the study are included in the article/Supplementary material, further inquiries can be directed to the corresponding authors.
