# Supplementary figures and images for: The impact of cerebral oxygen saturation monitoring on perioperative neurocognitive disorders: a meta-analysis and economic analysis
Source: Front Med (Lausanne). 2026 Jan 23;13:1677218. doi: 10.3389/fmed.2026.1677218 (PMC12876211; doi:10.3389/fmed.2026.1677218)

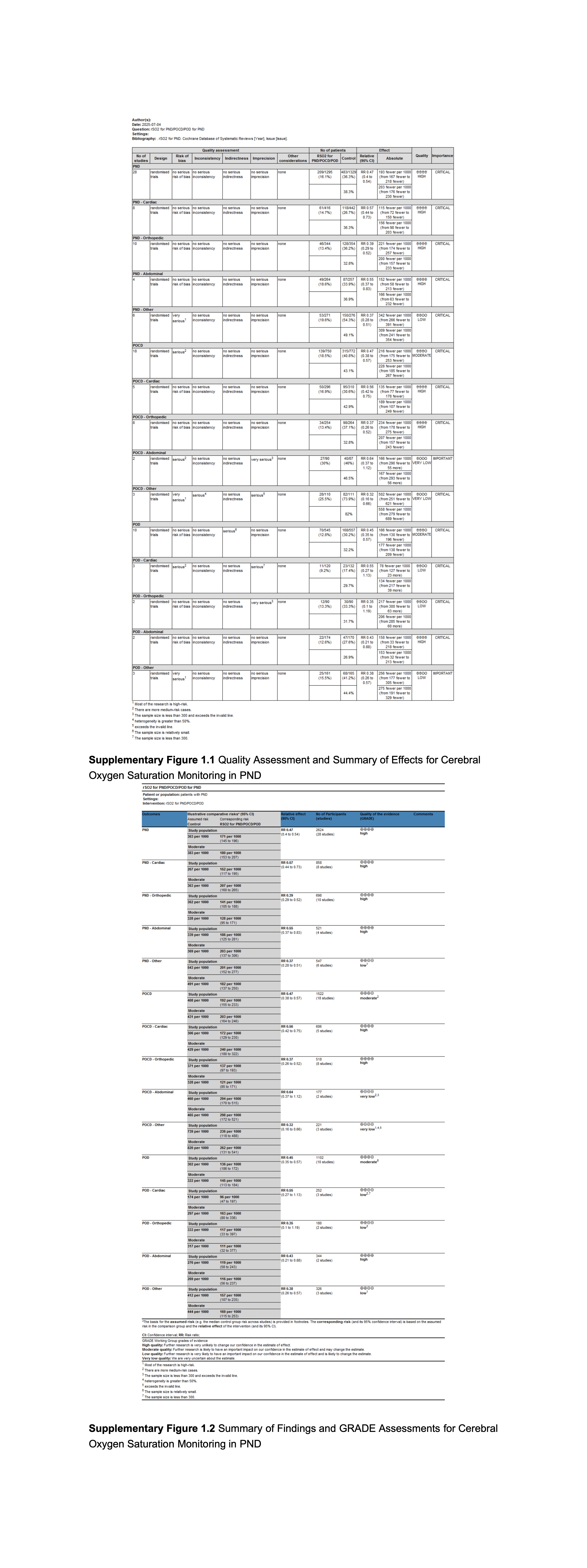

Supplement: Supplementary file 1 [file Image_1.JPEG]
