# Supplementary material for: The impact of cerebral oxygen saturation monitoring on perioperative neurocognitive disorders: a meta-analysis and economic analysis
Source: Front Med (Lausanne). 2026 Jan 23;13:1677218. doi: 10.3389/fmed.2026.1677218 (PMC12876211; doi:10.3389/fmed.2026.1677218)
Supplement: Supplementary file 2 [file Table_1.DOCX]

**Supplementary Table 1** Risk of Bias Assessment of Included Studies Using the RoB 2 Tool

| First Author (Year) | Randomization process | Deviations from intended interventions | Missing outcome data | Measurement of the outcome | Selection of the reported result | Overall Bias |
| --- | --- | --- | --- | --- | --- | --- |
| Wang L [19] (2020) | Low risk | Low risk | Low risk | Low risk | Low risk | Low risk |
| Cheng L [20] (2021) | Low risk | Low risk | Low risk | Low risk | Low risk | Low risk |
| Chen YH [21] (2020) | Low risk | Low risk | Low risk | Low risk | Low risk | Low risk |
| Yan LJ [22] (2020) | Low risk | High risk | Low risk | Some concerns | Low risk | High risk |
| Teng PL [23] (2020) | Low risk | Low risk | Low risk | Low risk | High risk | High risk |
| Xu QR [24] (2021) | Low risk | Low risk | Low risk | Low risk | Low risk | Low risk |
| Chen ZQ [25] (2021) | Low risk | Low risk | Low risk | Some concerns | High risk | High risk |
| Su XZ [26] (2021) | Low risk | Some concerns | Low risk | Low risk | Low risk | Some concerns |
| Wang JY [27] (2022) | Low risk | Low risk | Low risk | Low risk | Low risk | Low risk |
| Ballard [28] (2012) | Low risk | Low risk | Low risk | Low risk | Low risk | Low risk |
| Colak [8] (2014) | Low risk | Low risk | Low risk | Low risk | Low risk | Low risk |
| Murkin [29] (2007) | Low risk | Low risk | Low risk | Low risk | Low risk | Low risk |
| Slater [30] (2009) | Low risk | Low risk | Low risk | Low risk | Low risk | Low risk |
| Trafidlo [31] (2015) | Low risk | Low risk | Low risk | Low risk | Low risk | Low risk |
| Murniece [32] (2019) | Low risk | Low risk | Low risk | Low risk | Low risk | Low risk |
| Uysal [33] (2019) | Low risk | Low risk | Low risk | Low risk | Low risk | Low risk |
| Chen YJ [34] (2022) | Some concerns | Low risk | Low risk | Some concerns | Low risk | Some concerns |
| Gao Y [35] (2022) | Some concerns | Low risk | Low risk | Low risk | Low risk | Some concerns |
| Min XZ [36] (2019) | Low risk | Low risk | Low risk | Some concerns | Low risk | Some concerns |
| Wang X [37] (2023) | Low risk | Low risk | Low risk | Some concerns | High risk | High risk |
| Sahan [38] (2017) | Low risk | Low risk | Low risk | Low risk | Low risk | Low risk |
| Yang S [39] (2021) | Low risk | Low risk | Low risk | Low risk | Low risk | Low risk |
| Liang RR [40] (2020) | High risk | Low risk | Low risk | Some concerns | Low risk | High risk |
| Liu YL [41] (2017) | Some concerns | Low risk | Low risk | Low risk | High risk | High risk |
| Lin Y [42] (2019) | Low risk | Low risk | Low risk | Low risk | Low risk | Low risk |
| Song HJ [43] (2021) | Low risk | Low risk | Low risk | Some concerns | High risk | High risk |
| Casati [44] (2005) | Low risk | Low risk | Low risk | Low risk | Low risk | Low risk |
| Hekimoglu [45] (2023) | Some concerns | Low risk | Low risk | Low risk | Low risk | Some concerns |

Abbreviations: RoB, risk of bias. Assessment based on the Cochrane RoB 2.0 tool.
Low risk: Indicates low risk of bias; Some concerns: Indicates potential bias; High risk: Indicates high risk of bias.

Overall Bias reflects the highest level of concern across domains.
