# Supplementary material for: The impact of cerebral oxygen saturation monitoring on perioperative neurocognitive disorders: a meta-analysis and economic analysis
Source: Front Med (Lausanne). 2026 Jan 23;13:1677218. doi: 10.3389/fmed.2026.1677218 (PMC12876211; doi:10.3389/fmed.2026.1677218)
Supplement: Supplementary file 3 [file Table_2.DOCX]

**Supplementary Table 2** Subgroup Meta-Analysis Results by Assessment Tool, Follow-up Time, and rScO_2_ intervention threshold for PND, POCD, and POD

| Grouping Method | Outcome | Subgroup | No. of Studies | RR (95% CI) | *I* ^2^ (%) | *P* _h_ |
| --- | --- | --- | --- | --- | --- | --- |
| Scale | PND | Overall | 28 | 0.47 (0.41~0.54) | *2.8* | *0.442* |
|  |  | MMSE | 12 | 0.46 (0.36~0.60) | 41.8 | 0.063 |
|  |  | MoCA | 6 | 0.43 (0.29~0.65) | 0.0 | 0.958 |
|  |  | DRS | 2 | 0.50 (0.18~1.41) | 55.1 | 0.136 |
|  |  | CAM | 6 | 0.39 (0.28~0.54) | 0.0 | 0.665 |
|  |  | CAM-ICU | 2 | 0.52 (0.32~0.84) | 0.0 | 0.918 |
|  | POCD | Overall | 18 | 0.47 (0.39~0.57) | 16.3 | 0.259 |
|  |  | MMSE | 12 | 0.46 (0.36~0.60) | 41.8 | 0.063 |
|  |  | MoCA | 6 | 0.43 (0.29~0.65) | 0.0 | 0.958 |
|  | POD | Overall | 10 | 0.45 (0.35~0.57) | 0.0 | 0.618 |
|  |  | DRS | 2 | 0.50 (0.18~1.41) | 55.1 | 0.136 |
|  |  | CAM | 6 | 0.39 (0.28~0.54) | 0.0 | 0.665 |
|  |  | CAM-ICU | 2 | 0.52 (0.32~0.84) | 0.0 | 0.918 |
| Follow-up time | PND | Overall | 28 | 0.47 (0.41~0.54) | 2.8 | 0.442 |
|  |  | 1~3 day | 4 | 0.36 (0.22~0.58) | 0.0 | 0.794 |
|  |  | 4～7 day | 20 | 0.48 (0.40~0.57) | 16.7 | 0.246 |
|  |  | 1 month | 2 | 0.55 (0.30~1.03) | 0.0 | 0.550 |
|  |  | 3 month | 2 | 0.30 (0.15~0.59) | 0.0 | 0.916 |
|  | POCD | Overall | 18 | 0.47 (0.39~0.57) | 16.3 | 0.259 |
|  |  | 1~3 day | 1 | 0.36 (0.10~1.33) | NR | NR |
|  |  | 4～7 day | 13 | 0.47 (0.37~0.60) | 31.6 | 0.131 |
|  |  | 1 month | 2 | 0.55 (0.30~1.03) | 0.0 | 0.550 |
|  |  | 3 month | 2 | 0.30 (0.15~0.59) | 0.0 | 0.916 |
|  | POD | Overall | 10 | 0.45 (0.35~0.57) | 0.0 | 0.618 |
|  |  | 1~3 day | 3 | 0.35 (0.21~0.60) | 0.0 | 0.597 |
|  |  | 4～7 day | 7 | 0.48 (0.36~0.63) | 0.0 | 0.518 |
| rScO_2_ intervention threshold | PND | Overall | 27 | 0.48 (0.42~0.55) | 0.9 | 0.450 |
|  |  | > 90% | 3 | 0.49 (0.33~0.72) | 0.0 | 0.941 |
|  |  | 80%~90% | 18 | 0.45 (0.37~0.53) | 5.1 | 0.394 |
|  |  | <80% | 6 | 0.59 (0.40~0.86) | 8.1 | 0.364 |
|  | POCD | Overall | 18 | 0.48 (0.40~0.59) | 13.8 | 0.292 |
|  |  | > 90% | 1 | 0.52 (0.25~1.07) | NR | NR |
|  |  | 80%~90% | 12 | 0.44 (0.36~0.55) | 7.8 | 0.369 |
|  |  | <80% | 4 | 0.64 (0.44~0.94) | 6.2 | 0.362 |
|  | POD | Overall | 10 | 0.45 (0.35~0.57) | 0.0 | 0.618 |
|  |  | > 90% | 2 | 0.47 (0.29~0.76) | 0.0 | 0.786 |
|  |  | 80%~90% | 6 | 0.44 (0.31~0.62) | 16.4 | 0.308 |
|  |  | <80% | 2 | 0.28 (0.08~0.96) | 0.0 | 0.471 |

Abbreviations: RR, relative risk; CI, confidence interval; *I²*, heterogeneity statistic; *P_h_*, p-value for heterogeneity test; MMSE, Mini-Mental State Examination; MoCA, Montreal Cognitive Assessment; CAM, Confusion Assessment Method; DRS, Delirium Rating Scale; NR, not reported.
Data are grouped by neurocognitive assessment tool, follow-up duration, and rScO_2_ intervention threshold level.

The rScO_2_ intervention threshold refers to the rScO_2_ value (percentage of baseline) used in each study to initiate intraoperative corrective measures.
