# Supplementary material for: The impact of cerebral oxygen saturation monitoring on perioperative neurocognitive disorders: a meta-analysis and economic analysis
Source: Front Med (Lausanne). 2026 Jan 23;13:1677218. doi: 10.3389/fmed.2026.1677218 (PMC12876211; doi:10.3389/fmed.2026.1677218)
Supplement: Supplementary file 4 [file Table_3.DOCX]

**Supplementary Table 3** Operational definitions of POCD/POD in the 28 included RCTs

| The first author (Year) | Cognitive tool | Impairment threshold | Latest assessment | Intervention description |
| --- | --- | --- | --- | --- |
| Wang L [19] (2020) | CAM | NR | POD 7 d | Increasing arterial carbon dioxide partial pressure (PaCO₂), increasing inhaled oxygen concentration, adjusting head position, elevating hemoglobin (Hb) levels, and maintaining body temperature. |
| Cheng L [20] (2021) | CAM | Positive for CAM | POD 3 d | Bronchoscopy tube calibration Increase FiO₂, suction sputum, lower Ppeak, and administer bronchodilators; Blood pressure was controlled within ±20% of the baseline using norepinephrine or ephedrine. Add propofol to deepen anesthesia (BIS≥40); Check blood and qi to correct the internal environment. |
| Chen YH [21] (2020) | CAM | CAM > 22 | POD 7 d | Interventions included increasing arterial CO₂ content, adjusting the endotracheal tube position, raising blood pressure with fluids or vasopressors (ephedrine, phenylephrine), modulating anaesthetic depth, lowering body temperature and transfusing blood as required. |
| Yan LJ [22] (2020) | CAM | NR | POD 3 d | Increase blood pressure (MAP). |
| Teng PL [23] (2020) | CAM | CAM > 22 | POD 5 d | Verified probe position, adjusted anaesthesia depth, raised PaCO₂ to 45-55 mmHg by reducing VT or RR, and restored MAP within 20 % of baseline with fluids and norepinephrine. |
| Xu QR [24] (2021) | MoCA | MoCA Z ≥ 1.96 | POCD 7 d | Continuous central venous norepinephrine and supplemental fluids or bolus ephedrine/atropine as needed. |
| Chen ZQ [25] (2021) | MMSE | MMSE Z ≥ 1.96 | POCD 4 d | Instructed to deepen breathing, given increased FiO₂, and BP was raised with ephedrine or phenylephrine. |
| Su XZ [26] (2021) | MoCA | NR | POCD 5 d | Anaesthetists raised BP, increased FiO₂ and adjusted Hb level. |
| Wang JY [27] (2022) | CAM | CAM > 22 | POD 7 d | Head position was adjusted, catheter position checked, FiO₂ increased, PETCO₂ slightly raised, MAP augmented with fluids, and dobutamine. |
| Ballard [28] (2012) | MMSE | MMSE ISPOCD Z > 1.96 | POCD 3 mo | MAP was restored to within 10 % of baseline with fluids/inotropes, FiO₂ increased to 50–100 %, EtCO₂ raised to 5–6 %, and transfusion considered if Hb <9 g/dL, until rSO₂ returned to baseline. |
| Colak [8] (2014) | MMSE | MMSE ↓ ≥3 pts or CTT1/GP ↓ ≥1 SD | POCD 7 d | MAP/CO/pump flow were increased, FiO₂ raised, PaCO₂ elevated, depth or temperature lowered, and transfusion given if Hct <22 %. |
| Murkin [29] (2007) | MMSE | NR | POCD 1 mo | Pump flow was increased to 2.5 L/min/m², MAP raised to ≥60 mmHg with phenylephrine, PaCO₂ normalized to 40 mmHg, and FiO₂/pulsatile perfusion or propofol bolus added if needed. |
| Slater [30] (2009) | DRS | DRS ↓ ≥ 1 SD | POD 7 d | Repositioning of the head or perfusion cannulae; increasing arterial carbon dioxide tension, increasing systemic arterial blood pressure, adjusting pump flow rate or anesthetic depth; reduction of temperature; vasodilation; or blood transfusion. |
| Trafidlo [31] (2015) | MMSE | MMSE ↓ ≥ 20% | POCD 1 mo | They focused mainly on improving the head positioning whenever ScrO₂ declined by 20% from the baseline. When SpO₂ declines were noted, observers improved the orientation of pulse oximeter sensor. If this intervention was not effective the increase of FiO₂ took place. When the changes of MAP ±20% of baseline were found, the observers suited the supply of drug intravenous infusion (totally intravenous anesthesia) and the insertion of fluid volume. |
| Murniece [32] (2019) | MoCA | MoCA ↓ | POCD 2 d | Verify neutral head position, give 5–20 mg ephedrine to raise MAP, optimize SaO₂/PaCO₂/Hb, and exclude hyper-metabolic states. |
| Uysal [33] (2019) | MMSE | NR | POCD 3 mo | Verify transience, correct pH/PaO₂/PaCO₂ to pH 7.3–7.45/PaO₂ > 80 mmHg/PaCO₂ 35–50 mmHg, deepen anesthesia if BIS>60, give fluid if CVP<8 mmHg, give vasoactive meds to target MAP 80–90 mmHg, give inotrope for CI>2.0 L/min/m² if SvO₂ < 70%, transfuse to Hct>25% if Hct<20% (or consider if 20–25%), and during CPB maintain CPP 70–80 mmHg & flow >2.0 L/min/m², inform surgeon, consider brief antegrade cerebral perfusion or terminate HCA/RCP. |
| Chen YJ [34] (2022) | MMSE | NR | POCD 7 d | Head position adjustment, increasing inhaled oxygen concentration, boosting blood volume or applying low-dose vasoactive drugs. |
| Gao Y [35] (2022) | DRS | DRS ≥18 | POD 2 d | Suspend the surgical operation and maintain the effective circulating blood volume by measures such as raising blood pressure (administering methamine) or bilateral pulmonary ventilation. |
| Min XZ [36] (2019) | MMSE | MMSE ↓ ≥ 2 | POCD 7 d | The intervention was carried out successively through the head position, the position of the superior and inferior aortic vena cava intubation, mean arterial pressure, arterial SpO₂, arterial blood carbon dioxide, hemoglobin, and mixed blood oxygen saturation. |
| Wang X [37] (2023) | MMSE | NR | POCD 7 d | Maintaining body temperature, increasing hemoglobin (Hb), raising arterial partial pressure of carbon dioxide (PaCO₂), and increasing inhaled oxygen concentration should be adopted. If necessary, the clarity of the surgical field should be reduced and hypertension should be raised to ensure surgical safety. |
| Sahan [38] (2017) | MMSE | MMSE ↓ ≥ 1 SD | POCD 7 d | The algorithm to correct intraoperative cerebral desaturation proposed by Denault was used… first step was to rule out any mechanical obstruction… then to increase mean arterial pressure and to verify systemic oxygenation… normalize PaCO₂, optimize hemoglobin level, evaluate cardiac function and finally to decrease cerebral metabolic rate of oxygen. |
| Yang S [39] (2021) | MoCA | MoCA Z ≥ 1.96 | POCD 7 d | Correct MAP, PETCO₂, Hb, SpO₂ in sequence (for raising blood pressure, adjusting ventilation, increasing FiO₂, blood transfusion, etc.) |
| Liang RR [40] (2020) | MoCA | MoCA ↓ | POCD 7 d | Depending on the situation, brain-protective drugs, ice caps and other treatments that lower brain metabolism can be given. |
| Liu YL [41] (2017) | CAM-ICU | NR | POD 7 d | Confirm accurate electrode placement and head position; keep MAP within ±10 % of the pre-induction value via fluids, blood, or vasopressors; titrate anesthetic depth and FiO₂; set ventilation to achieve mild hypercapnia (PaCO₂ just above 40 mmHg); and continuously monitor and correct electrolytes to maintain normal levels. |
| Lin Y [42] (2019) | CAM-ICU | NR | POD 7 d | Head position, increase CO₂, raise blood pressure (m-hydroxyamine/norepinephrine), strengthen the heart (epinephrine), deepen anesthesia (BIS≤50), add pump flow, FiO₂, and receive blood transfusion. |
| Song HJ [43] (2021) | MoCA | MoCA Z > 2 | POCD 7 d | Measures such as adjusting the position of the head, regulating blood pressure, increasing FiO₂, and adjusting respiratory parameters to increase PETCO₂. |
| Casati [44] (2005) | MMSE | MMSE ↓ ≥ 2 | POCD 7 d | First, check the ventilator, circuit and head position. Simultaneously increase FiO₂, raise ETco₂ to >35 mmHg, and increase the blood pressure with 250 mL of hydroxyethyl starch and ethylphenyfline (when SBP≤90 mmHg). If recovery does not occur within 60 seconds, the second step is to intravenously inject propofol to reduce cerebral oxygen consumption until rSO₂ returns to the target range. |
| Hekimoglu [45] (2023) | MMSE | MMSE score of < 23 | POCD 5 d | First, check and adjust the position of the head and neck, then increase MAP to improve the overall oxygen supply. Subsequently, successively adjust PaCO₂ and hemoglobin to the normal range, assess cardiac function, and take measures to lower brain metabolism if necessary. |

Abbreviations: CAM, Confusion Assessment Method; CAM-ICU, Confusion Assessment Method for the ICU; DRS, Delirium Rating Scale; MMSE, Mini-Mental State Examination; MoCA, Montreal Cognitive Assessment; NR, threshold not reported in the original paper; SD, standard deviation; POD, postoperative delirium; POCD, postoperative cognitive dysfunction; Latest assessment, latest post-operative day/month at which cognition was evaluated; Impairment threshold, cut-off used to define cognitive decline (NR indicates the exact value was not provided).
